# Many but small HIV-1 non-B transmission chains in the Netherlands

**DOI:** 10.1097/QAD.0000000000003074

**Published:** 2021-10-05

**Authors:** Daniela Bezemer, Alexandra Blenkinsop, Matthew Hall, Ard van Sighem, Marion Cornelissen, Els Wessels, Jeroen van Kampen, Thijs van de Laar, Peter Reiss, Christophe Fraser, Oliver Ratmann

**Affiliations:** a Stichting HIV Monitoring, Amsterdam, The Netherlands; b Department of Mathematics, Imperial College London, London; c Department of Global Health, Amsterdam University Medical Centers, University of Amsterdam and Amsterdam Institute for Global Health and Development, Amsterdam, The Netherlands; d Oxford Big Data Institute, University of Oxford, Oxford, UK; e Laboratory of Clinical Virology, Department of Medical Microbiology, Academic Medical Center of the University of Amsterdam, Amsterdam; f Department of Medical Microbiology, Leiden University Medical Center, Leiden; g Erasmus MC, Rotterdam; h Department of Donor Medicine Research, laboratory of Blood-borne Infections, Sanquin Research; i Department of Medical Microbiology, Onze Lieve Vrouwe Gasthuis, Amsterdam, The Netherlands.

**Keywords:** HIV-1, introduction, migrants, MSM, phylogeny, phylogeographic origin, risk-group, spread, sub-epidemics, subtypes, transmission chains, unobserved size distribution

## Abstract

The aim of this study was to investigate introductions and spread of different HIV-1 subtypes in the Netherlands.

**Design:**

We identified distinct HIV-1 transmission chains in the Netherlands within the global epidemic context through viral phylogenetic analysis of partial HIV-1 polymerase sequences from individuals enrolled in the ATHENA national HIV cohort of all persons in care since 1996, and publicly available international background sequences.

**Methods:**

Viral lineages circulating in the Netherlands were identified through maximum parsimony phylogeographic analysis. The proportion of HIV-1 infections acquired in-country among heterosexuals and MSM was estimated from phylogenetically observed, national transmission chains using a branching process model that accounts for incomplete sampling.

**Results:**

As of 1 January 2019, 2589 (24%) of 10 971 (41%) HIV-1 sequenced individuals in ATHENA had non-B subtypes (A1, C, D, F, G) or circulating recombinant forms (CRF01AE, CRF02AG, CRF06-cpx). The 1588 heterosexuals were in 1224, and 536 MSM in 270 phylogenetically observed transmission chains. After adjustments for incomplete sampling, most heterosexual (75%) and MSM (76%) transmission chains were estimated to include only the individual introducing the virus (size = 1). Onward transmission occurred mostly in chains size 2–5 amongst heterosexuals (62%) and in chains size at least 10 amongst MSM (64%). Considering some chains originated in-country from other risk-groups, 40% (95% confidence interval: 36–44) of non-B-infected heterosexuals and 62% (95% confidence interval: 49–73) of MSM-acquired infection in-country.

**Conclusion:**

Although most HIV-1 non-B introductions showed no or very little onward transmission, a considerable proportion of non-B infections amongst both heterosexuals and MSM in the Netherlands have been acquired in-country.

## Introduction

The WHO has the ambition to end HIV transmission this decade [1]. With this aim, it is essential for countries to understand where HIV infections were acquired, and who they affect. In Western European countries, an increasing proportion of newly HIV-1 diagnosed persons are infected with non-B subtypes [2–6]. In the Netherlands, this concerns about a quarter of all people receiving care. In 71% of cases, these are foreign-born individuals, of whom 70% are from sub-Saharan Africa. The level of introductions and national transmission is unknown. The aMASE survey study among immigrants across HIV clinics in Europe estimated that 45% (95% confidence interval: 39–52) of infections among people from sub-Saharan Africa were acquired postmigration to Western Europe [7]. In contrast, a molecular phylogenetic study from Europe suggests that only nearly 20% of non-B infections among people from sub-Saharan Africa acquired their infection postmigration [2]. These estimates are quite different; however, the phylogenetic study had not accounted for individuals that were not sequenced, potentially introducing sampling bias in these estimates.

To obtain more insight into the transmission dynamics of non-B subtypes, we reconstructed partially observed transmission chains through phylogenetic analysis of nationally collected HIV-1 *polymerase* (*pol*) nucleotide sequences, and then estimated the proportion of in-country HIV acquisitions amongst heterosexuals and MSM while accounting for incomplete sequence sampling of these risk groups. The sequences were obtained from the ATHENA national observational HIV cohort, and combined with *pol* sequences publicly available from the Los Alamos National Lab (LANL) HIV database [4,8]. As subtypes evolve differently amongst different risk groups, and some people have their virus sequenced many years after infection, we avoid phylogenetic clustering analysis with cut-offs on bootstrap values or patristic distances in phylogenetic trees [9–12]. Instead, the LANL sequences provided the phylogeographic context to identify distinct viral phylogenetic subgraphs associated with the Netherlands. We then interpreted each subgraph as evidence of a distinct national transmission chain resulting from a single introduction. This made it possible to estimate the number, spread, as well as the origin of introductions by transmission group of distinct non-B transmission chains, and for comparison, subtype B transmission chains in the Netherlands. Using a branching process model that adjusts for incomplete observations our results provide insight regarding the proportion of HIV-1 infections that are acquired in-country, both for heterosexual infected individuals and MSM.

## Materials and methods

### ATHENA cohort

The ATHENA national observational HIV cohort includes pseudonymized demographic and clinical data collected from HIV-positive individuals receiving care in one of the 24 HIV treatment centres in the Netherlands since 1996, excluding approximately 1.5% who opt-out of their data being collected [4]. Population-based nucleotide HIV-1 partial *pol* gene sequences are obtained as part of routine screening for antiretroviral drug resistance, either before start of antiretroviral therapy or at time of virological failure [13]. Data on the most likely mode of HIV infection and assumed geographic region of infection are self-reported at time of entry into HIV care.

### HIV-1 sequence selection, alignment and subtyping

The study population comprised individuals in the ATHENA cohort infected before 1 January 2019 with at least one available HIV-1 *pol* RNA sequence with a minimum length of 750 nucleotides by 1 June 2020. If more than one sequence was available, the first sequence was used for analysis. *R* and the *ape* package were used to process sequences for phylogenetic analysis [14]. Sequences were aligned to the reference genome HXB2 [15], using *VIRULIGN*[16]. Subtypes were assigned using *COMET*[17], and when required checked and assigned using *REGA*[18].

### International epidemic context

To characterize the international epidemic context of sequences obtained from our study population, background sequences were retrieved by subtype from the LANL HIV database on 18 February 2021 [8]. LANL sequences were included into the background sequence data if they overlapped with the ATHENA sequences in at least 1000 nucleotides, had country and year of sampling reported, and were not from the Netherlands. Only one sequence per person was included. Accession numbers are reported in Supplementary Table S1. In addition, ATHENA sequences from people on Curaçao were added as background sequences.

### Phylogeny construction

For each subtype, aligned background sequences were merged with the alignment of the study sequences using the reference sequence HXB2. Sequences from the closest other subtype in the combined data set were included as outgroups. For every subtype-specific alignment, major resistance conferring sites were deleted [19]. Phylogenetic trees were built in FastTree v2.1.8 [20] and rooted at the outgroup.

### Viral phylogenetic estimation of Dutch subgraphs by transmission risk group

**Fig. 1:**
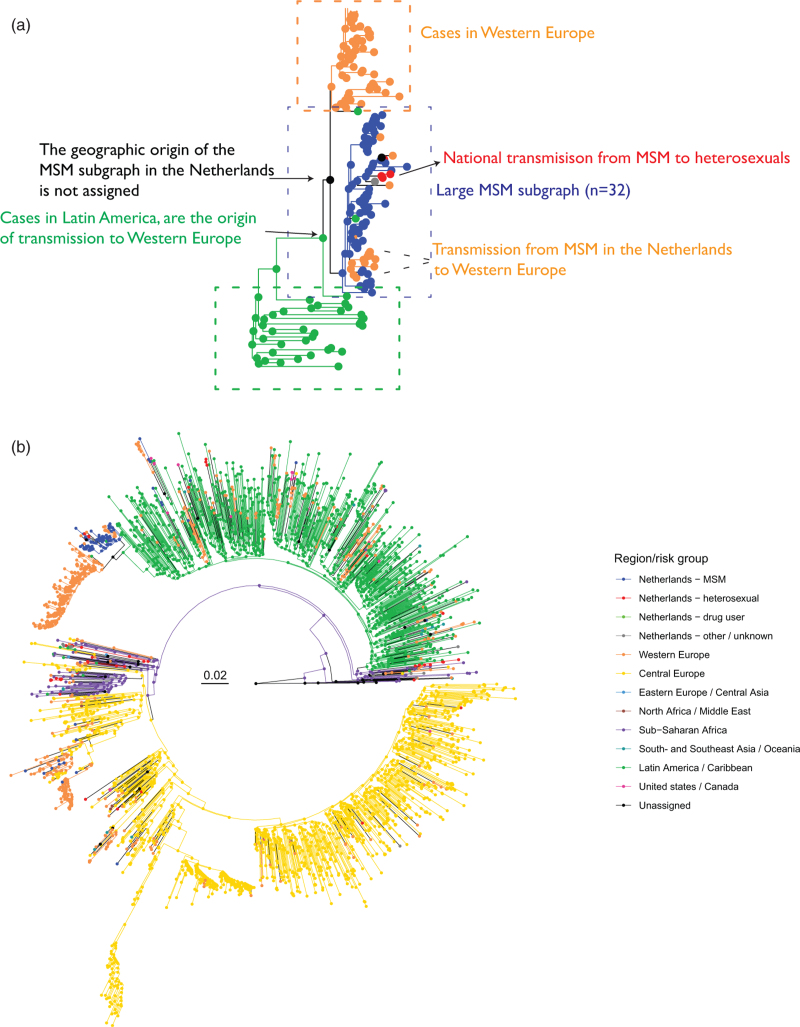
(a) Methodological sketch of phylogeographic analysis to identify HIV-1 transmission chains associated with either MSM or heterosexuals in the Netherlands. (b) Reconstructed viral phylogeny of subtype F partial HIV-1 pol sequences of participants enrolled in the Dutch national ATHENA cohort, and the corresponding collection of viral sequences from the Los Alamos National Lab HIV database.

From the reconstructed phylogenies, we identified phylogenetic subgraphs associated with national transmission amongst either heterosexuals or MSM. First ATHENA sequences were associated with states according to the transmission risk group (heterosexual, MSM, drug users, other/unknown) of the corresponding sampled individual. The state ‘other/unknown’ includes individuals infected through blood transfusion, vertical transmissions, accidental/occupational exposure and unknown routes of transmission. States associated with sequences from the LANL HIV database were based on the geographic region of the sample (states: Central Europe, Eastern Europe, Western Europe, Latin America and the Caribbean, North Africa and the Middle East, sub-Saharan Africa, United States and Canada, South- and Southeast Asia and Oceania, and Suriname and Curaçao, Supplementary Table S1). Second, ancestral states of all internal nodes in the phylogeny were estimated with the maximum parsimony ancestral state reconstruction algorithm implemented in *phyloscanner* v1.8.0 [21] (Supplementary Text, section 1). Then, likely transmission chains associated with either MSM or heterosexuals in the Netherlands were identified as phylogenetic subgraphs (of size ≥1) that had a common state change occurring at the branch leading to the node representing their most recent common ancestor (see Fig. 1a).

Viral phylogenetic lineages were associated to geographic regions based on the sampling location of sequenced individuals and transmission category in the Netherlands with maximum parsimony. External dots represent the sequenced samples. Colours of internal dots and branches represent the estimated ancestral location of the viral lineages.

The phylogeographic origin of a Dutch phylogenetically identified transmission chain was estimated as the state of the viral lineage ancestral to its root, which was one of the geographic regions attributed to LANL sequences, another Dutch transmission risk group or undefined when two states were equally parsimonious.

### Statistical analyses

The number, origin and size of the phylogenetically identified transmission chains were determined by summing these statistics across the subtype-specific phylogenies. We focused on the four largest transmission categories (*r*) in the Netherlands: non-B heterosexuals, non-B MSM, subtype B heterosexuals and subtype B MSM. Ninety-five percent confidence intervals of these statistics were obtained from 100 replicate analyses on bootstrap sequence alignments, and are abbreviated as 95%BS-CI. As a result, confidence intervals do not necessarily include the central estimate.

In a fully sampled population, every phylogenetically identified transmission chain would correspond to the actual transmission chain within a particular observation period, and comprise one introduction possibly followed by onward in-country transmission. Some introductions will have occurred from other in-country risk groups, and so, the proportion of HIV-1 infections acquired in the Netherlands in category *r* can be expressed as


(1) $$p_{r}=1-(\alpha_{r}E_{r}^{*})/N_{r}^{*}$$

where $E_{r}^{*}$ is the number of transmission chains in category *r*, *α*_*r*_ the proportion of chains that had their origin assigned to outside the Netherlands and $N_{r}^{*}$ is the total number of individuals in the transmission chains. However, it is not obvious whether unsampled infections in the Netherlands are part of unobserved transmission chains or represent additional infections in partially observed chains. This makes it necessary to account for incomplete sampling when analysing phylogenetically observed transmission chains. The likelihood of the observed chain size distribution (*x*) was quantified under a branching process model with Negative Binomial offspring distribution to describe the unobserved size distribution of transmission chains (*z*), assuming that infected individuals were sampled at random [22]. The model is specified by the expected number of infected individuals under the offspring distribution, the dispersion parameter under the offspring distribution and the sampling probability (the ratio of individuals with a sequence divided by the total number of HIV-1 positive individuals in ATHENA). Using a Bayesian framework (Supplementary Text, section 2), we sampled from the posterior predictive distribution of complete transmission chains $z_{r}^{*}$, calculated $N_{r}^{*}$ and $E_{r}^{*}$, and estimated the proportion of HIV-1 infections acquired in the Netherlands in category *r* through Eq. (1). Sampling-adjusted results are reported by the median estimates and corresponding 95% credible intervals (95% CI). The model does not account for incomplete sampling of infections occurring outside the Netherlands based on the rationale that the sequence of only one external individual separating any two Dutch transmission chains needs to be sampled in order to separate distinct Dutch transmission chains into distinct phylogenetic subgraphs.

### Comparison to estimates from the aMASE survey

Phylogenetic estimates on the proportion of HIV-1 infections acquired in the Netherlands amongst MSM and heterosexuals with non-B subtypes were compared with estimates from the aMASE cross-sectional study among HIV-positive migrants. The study was conducted from July 2013 to July 2015 in 57 clinics in nine European countries. Postmigration HIV acquisition was estimated from electronic questionnaire and clinical data [7].

### Ethics approval

At initiation, the ATHENA cohort was approved by the institutional review board of all participating HIV treatment centres. People entering HIV care receive written material about participation in the ATHENA cohort and are informed by their treating physician on the purpose of data collection, thereafter they can consent verbally or elect to opt-out. Data are pseudonymised before being provided to investigators and may be used for scientific purposes. A designated quality management coordinator safeguards compliance with the European General Data Protection Regulation [4].

## Results

### Study population

As of 1 January 2019, the ATHENA cohort included 26,881 HIV-1 positive individuals registered in the Netherlands (Table 1). Of these, 10 971 (41%) had a partial *pol* sequence available in this study, of whom 8382 (76%) were subtype B, and 2589 (24%) belonged to eight non-B subtypes or circulating recombinant forms (CRFs) included in this study. A remaining 266 individuals had undefined or other minority (recombinant) subtypes (Supplementary Information SI.2), and were not considered further. Of the 2589 non-B HIV-1 infections included in this study, 417 (16%) were subtype A1, 615 (24%) subtype C, 111 (4%) subtype D, 93 (4%) subtype F, 173 (7%) subtype G, 338 (13%) CRF01AE, 770 (30%) CRF02AG and 72 (3%) CRF06-cpx.

**Table 1:** Characteristics of the study population, sequenced HIV-1 infected participants of the ATHENA study, the Netherlands, and the number of added international background sequences.

		Sequences in study		
	All	Total	non-B subtypes	subtype B
Number of ATHENA participants	26 881	10 971	2589	8382
Transmission risk group				
MSM	15 882 (59%)	6949 (63%)	536 (21%)	6413 (77%)
Heterosexual	7608 (28%)	2932 (27%)	1588 (61%)	1344 (16%)
Drug users	785 (3%)	236 (2%)	27 (1%)	209 (2%)
Other and unknown	2606 (10%)	854 (8%)	438 (17%)	416 (5%)
Sampling year median (IQR)	–	2008 (2005–2012)	2009 (2005–2012)	2008 (2005–2011)
Number of added international background sequences^a^	–	139 858	66 181	73 677
Sampling year median (IQR)	–	2009 (2006–2013)	2010 (2007–2013)	2009 (2005–2012)

a 139 633 sequences obtained from the LANL HIV database and 225 ATHENA sequences from people on Curaçao.

Of the 2589 non-B-infected individuals, 1588 (61%) were self-reported heterosexuals, 536 (21%) MSM and 465 (18%) reported other or unknown modes of HIV-1 transmission. Region of birth varied significantly among both transmission categories. Among heterosexuals, 20% were born in the Netherlands, 62% in sub-Saharan Africa, 6% in North Africa and Middle East, 4% in South- and Southeast Asia and Oceania, 3% in Suriname and Curaçao, 2% in Western-Europe and 3% elsewhere or unknown. Among MSM, 61% were born in the Netherlands, 8% in South- and Southeast Asia and Oceania, 7% in Western-Europe, 6% in sub-Saharan Africa, 5% in Latin America or the Caribbean, 4% in Suriname or Curaçao, 4% in North Africa and Middle East, and 5% elsewhere or unknown.

Of 6707 sequenced subtype B infections, a majority 77% was found among MSM and 16% among heterosexuals, where the majority was born in the Netherlands (heterosexuals 52%, MSM 70%). Supplementary Table S2 characterizes the ATHENA cohort and the subset of participants with identified subtype in further detail. Supplementary Table S3 characterizes the international background sequences by subtype and geographic region.

### Number and origin of phylogenetically observed transmission chains

Subtype-specific viral phylogenetic trees were constructed from 10 971 ATHENA sequences and 139 859 international sequences. Viral lineages in these trees were associated with nine world-geographic regions as well as the Dutch transmission categories MSM, heterosexual, drug users, other (see Methods) as shown in Supplementary Figures S1–S39, and illustrated in Fig. 1 for analysis of one the smaller trees (subtype F). See Supplementary Table S4 and Table S5, for results by subtype.

#### HIV-1 non-B subtypes

The 1588 persons infected heterosexually with non-B virus were part of 1224 (95%BS-CI: 1220–1270) observed, phylogenetically identified transmission chains (Table 2, panel ‘non-B subtypes’, ‘heterosexual’), indicating that a large number of distinct non-B transmission chains are co-circulating in the Netherlands. Among these phylogenetically observed transmission chains, an estimated 8% (95%BS-CI: 5–8) originated from Western Europe, 76% (95%BS-CI: 75–78) from sub-Saharan Africa, 11% (95%BS-CI: 11–13) from Southeast Asia and Oceania, and 2% (95%BS-CI: 2–3) from other world regions. Only 3% (95%BS-CI: 2–3) originated from national MSM transmission chains, and 1% (95%BS-CI: 0–1) from other national chains (Table 3).

**Table 2:** Number and size of phylogenetically observed and estimated actual transmission chains for HIV-1 subtype B and non-B subtypes amongst MSM and heterosexuals in the Netherlands.

	Analysis by transmission group and subtype in the Netherlands							
	Non-B subtypes				Subtype B			
	Phylogenetically observed transmission chains^a^		Estimated actual transmission chains^b^		Phylogenetically observed transmission chains^a^		Estimated actual transmission chains^b^	
	*N* (95% BS-CI)	% (95%BS-CI)	*N* (95% CI)	% (95% CI)	*N* (95% BS-CI)	% (95%BS-CI)	*N* (95% CI)	% (95% CI)
Heterosexuals								
Number of transmission chains	1224 (1220–1270)	100	2,581 (2403–2754)	100	856 (866–908)	100	1857 (1644–2063)	100
Number of individuals	1588	100	4121 (4121–4129)	100	1344	100	3487 (3487–3506)	100
Transmission chains size 1	1005 (994–1065)	82 (82–85)	1931 (1713–2150)	75 (70–79)	676 (687–736)	79 (79–82)	1414 (1200–1635)	76 (71–80)
Individuals in chains size 1	1005 (994–1065)	63 (63–67)	1,931 (1713–2151)	47 (42--52)	676 (687–736)	50 (51–55)	1414 (1200–1635)	41 (34–47)
Transmission chains size 2–5	211 (184–217)	17 (15–17)	572 (482–665)	22 (18–26)	161 (145–172)	19 (16–19)	340 (272–414)	18 (15–23)
Individuals in chains size 2–5	501 (38–515)	32 (28–32)	1530 (1298–1766)	37 (31–43)	420 (364–437)	31 (27–33)	942 (755–1143)	27 (22–33)
Transmission chains size 6–9	6 (1–8)	1 (0–1)	57 (43–73)	2 (2–3)	13 (5–15)	2 (1–2)	56 (42–71)	3 (2–4)
Individuals in chains size 6–9	40 (7–53)	3 (0–3)	403 (296–517)	10 (7–13)	100 (37–110)	7 (3–8)	398 (296–509)	11 (8–15)
Transmission chains size ≥10	2 (2–3)	0.2 (0.2–0.2)	19 (9–32)	1 (0–1)	6 (5–8)	1 (1–1)	44 (31–57)	2 (2–3)
Individuals in chains size ≥10	42 (23–59)	3 (1–4)	248 (114–438)	6 (3–11)	148 (126–184)	11 (9–14)	724 (469–1036)	21 (13–30)
MSM								
Number of transmission chains	270 (266–284)	100	518 (358–662)	100	2154 (2286–2397)	100	3857 (3285–4420)	100
Number of individuals	536	100	1226 (1225–1279)	100	6,413	100	14 662 (14 657–14 786)	100
Transmission chains size 1	209 (200–222)	77 (75–79)	392 (257–532)	76 (68–83)	1486 (1610–1723)	69 (70–72)	2680 (2249--3130)	70 (67–72)
Individuals in chains size 1	209 (200–222)	39 (37–41)	392 (257–532)	32 (22–43)	1486 (1610–1723)	23 (25–27)	2680 (2249–3130)	18 (15–21)
Transmission chains size 2–5	50 (45–56)	19 (16–20)	84 (51–118)	16 (11–22)	498 (492–539)	23 (21–23)	740 (613–872)	19 (17–21)
Individuals in chains size 2–5	122 (109–138)	23 (20–26)	235 (144–333)	19 (12–27)	1349 (1324–1458)	21 (21–23)	2093 (1731–2467)	14 (12–17)
Transmission chains size 6–9	3 (1–5)	1 (0–2)	16 (8–26)	3 (2–5)	76 (60–81)	4 (3–3)	158 (124–192)	4 (3–5)
Individuals in chains size 6–9	20 (8–36)	4 (1–7)	118 (59–187)	10 (5–15)	545 (421–579)	8 (7–9)	1,137 (896–1,389)	8 (6–9)
Transmission chains size ≥10	8 (7–10)	3 (3–4)	21 (14–28)	4 (2–7)	94 (86–100)	4 (4–4)	276 (248–304)	7 (6–8)
Individuals in chains size ≥ 10	185 (160–195)	35 (30–36)	477 (278–729)	39 (23–59)	3033 (2755–2955)	47 (43–46)	8759 (7873–9681)	60 (54–66)

a Central estimates of phylogenetically identified transmission chains were determined by summing these statistics across the subtype-specific phylogenies. 95% confidence intervals of these statistics were obtained from 100 replicate analyses on bootstrap sequence alignments. As a result, confidence intervals do not necessarily include the central estimate.

b Estimated actual transmission chains, results adjusted for incomplete observations.

**Table 3:** Estimated geographic origins of HIV-1 subtype B and non-B subtypes phylogenetically likely transmission chains amongst MSM and heterosexuals in the Netherlands.

Analysis by transmission group and subtype in the Netherlands				
	Non-B subtypes		Subtype B	
	Heterosexual	MSM	Heterosexual	MSM
	% (95%BS-CI)	% (95%BS-CI)	% (95%BS-CI)	% (95%BS-CI)
Phylogeographic origin of subgraphs (%)				
Europe - Central	1 (1–1)	2 (1–4)	1 (0–1)	3 (2–3)
Europe - West	8 (5–8)	22 (15–22)	19 (15–21)	43 (36–41)
Europe – East and Central Asia	0 (0–0)	1 (1–2)	0 (0–0)	0 (0–0)
Latin America, Caribbean	1 (0–1)	3 (2–5)	11 (9–13)	12 (11–14)
Netherlands- heterosexuals	–	4 (2–7)	–	3 (2–3)
Netherlands – drug users	0 (0–0)	0 (0–0)	3 (1–2)	0 (0–0)
Netherlands - MSM	3 (2–3)	–	50 (44–50)	–
Netherlands – other/unknown	1 (0–1)	0 (0–1)	1 (1–2)	0 (0–1)
North Africa and Middle East	0 (0–0)	1 (0–1)	0 (0–0)	0 (0–0)
North America	0 (0–1)	0 (0–1)	13 (14–22)	37 (38–44)
Southeast Asia and Oceania	11 (11–13)	41 (39–45)	0 (0–1)	1 (1–2)
Suriname and Curaçao	0 (0–0)	0 (0–0)	3 (2–4)	0 (0–1)
Sub-Saharan Africa	76 (75–78)	25 (23–30)	0 (0–0)	0 (0–0)
Proportion of total subgraphs with resolved origin	78 (78–82)	79 (74–83)	79 (72–78)	78 (72–76)
Proportion of total individuals included	79 (76–83)	73 (67–85)	79 (67–78)	78 (68–79)

Central estimates of phylogenetically identified transmission chains were determined by summing these statistics across the subtype-specific phylogenies. 95% confidence intervals of these statistics were obtained from 100 replicate analyses on bootstrap sequence alignments. As a result, confidence intervals do not necessarily include the central estimate.

The 536 non-B infections amongst MSM were part of 270 (95%BS-CI: 266–284) observed, phylogenetically identified transmission chains (Table 2, panel ‘non-B subtypes’, ‘MSM’). An estimated 22% (95%BS-CI: 15–22) originated from Western Europe, 25% (95%BS-CI: 23–30) from sub-Saharan Africa, 41% (95%BS-CI: 39–45) from Southeast Asia and Oceania, 8% (95%BS-CI: 6–10) from other world regions, 4% (95%BS-CI: 2–7) from heterosexuals in the Netherlands and 0 (95%BS-CI: 0–1) from other national chains (Table 3).

### Estimated transmission chains after accounting for incomplete sequence sampling

Accounting for incomplete sampling, we estimated more and larger transmission chains than phylogenetically observed (Table 2). The size of the estimated transmission chains was highly heterogeneous, with 75% (95% CI: 70–79) of chains among non-B heterosexuals and 76% (95% CI: 68–83) among non-B MSM being of size 1. The largest proportion of onwards transmitted heterosexuals occurred in transmission chains of size 2–5 [62% (95% CI: 52–72)], and amongst MSM in chains of size at least 10 [64% (95% CI: 45–81)]. Supplementary Table S6 summarizes the composition of large observed subgraphs by region of birth and region of self-reported infection. Supplementary Table S7 shows observed chain size distributions disaggregated by size and subtype.

#### Comparison to HIV-1 subtype B

For comparison, we characterized the number of subtype B introductions in the same manner (Table 2, panel ‘subtype B’). The persons infected heterosexually with subtype B were attributed to proportionally fewer and significantly larger transmission chains than heterosexual individuals infected with non-B subtypes. The phylogenetically inferred origins of subtype B transmission chains were significantly different to those inferred for non-B transmission chains, with an estimated 50% (95%BS-CI: 44–50) originating from national MSM transmission chains (Table 3). MSM infected with subtype B were also attributed to proportionally fewer and significantly larger transmission chains than MSM infected with non-B subtypes. The phylogenetically inferred origins of subtype B transmission chains among MSM were also significantly different, with more chains originating from Western Europe, North America and Latin America and the Caribbean, as compared to non-B transmission chains among MSM.

### Proportion of HIV-1 infections acquired in the Netherlands

After adjusting for HIV-1 infected individuals who were not sequenced, an estimated 62% (95% CI: 58–67) of non-B-infected heterosexuals represented a viral introduction in the estimated transmission chains, of whom 96% (95% CI: 94–97) had an estimated external origin (Table 3). Combining these estimates, we estimate that 40% (95% CI: 36–44) of non-B-infected heterosexuals in the Netherlands acquired HIV in-country. Considering non-B-infected MSM, an estimated 41% (95% CI: 28–53) of chains represented a viral introduction, of which 94% (95% CI: 90–96) had an external origin, and we estimate that 60% (95% CI: 49–73) of non-B-infected MSM acquired HIV in the Netherlands.

**Fig. 2:**
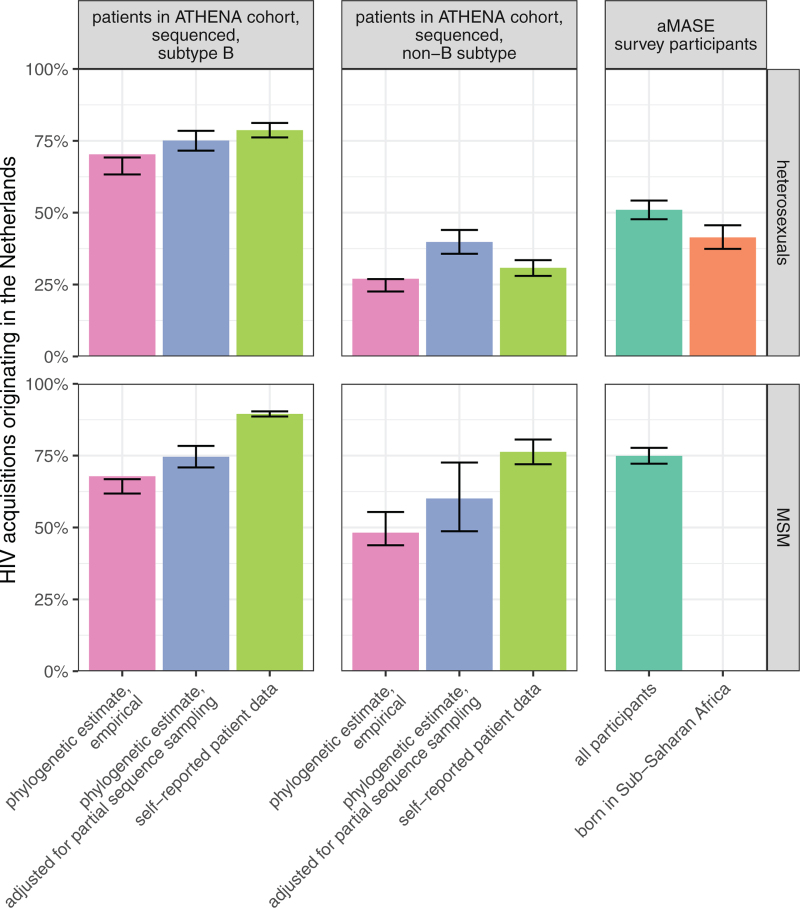
Estimated proportion of in-country HIV-1 acquisition by transmission group in the Netherlands.

Figure 2 compares the estimated proportion of HIV in-country acquisitions among heterosexuals and MSM with non-B virus to those with subtype B virus. For subtype B-infected heterosexuals in the Netherlands, after adjusting for incomplete sampling, an estimated 52% (95% CI: 46–58) represented a viral introduction, of which 48% (95% CI: 44–52) had a foreign origin. Combining these estimates, we find that an estimated 75% (95% CI: 72–79) of subtype B-infected heterosexuals acquired HIV in-country. Considering subtype B-infected MSM, an estimated 26% (95% CI: 22–30) represented a viral introduction in the unobserved complete transmission, of which 97% (95% CI: 96–98) had a foreign origin, and 75% (95% CI: 71–78) acquired HIV in-country.

Viral phylogeographic analyses were used to identify likely transmission chains among heterosexual and MSM participants of the Dutch national ATHENA cohort for each of the main circulating subtypes. Estimates of the sequence sampling fraction were used to predict sizes of the unobserved complete transmission chains. Estimates of the proportion of transmission chains originating from outside the Netherlands were used to estimate the proportion of in-country HIV acquisitions in the unobserved complete transmission chains. In comparison, phylogenetic empirical estimates are shown with 95% confidence intervals obtained from 100 replicate analyses on bootstrap sequence alignments, hence these do not necessarily include the central estimate. In addition, estimates from self-reported data on the likely location of acquisition, and a selection of estimates from the aMASE survey study among immigrants across HIV clinics in Europe are shown [7].

Thus, overall, the estimated proportions of in-country HIV acquisition were higher among subtype B-infected when compared to non-B-infected, especially so for heterosexuals. The estimated proportion of in-country transmission was larger for all risk groups and all subtypes after adjusting for incomplete sequence sampling (Fig. 2), and more consistent with estimates from self-reported data on the likely location of acquisition as well as relevant estimates from the aMASE survey study among immigrants across HIV clinics in Europe [7] than the unadjusted estimates.

## Discussion

We characterized the number, level of spread and region of origin of HIV-1 non-B introductions in the Netherlands by viral phylogenetic analysis, and compared our findings with those for HIV-1 subtype B infections. Our phylogenetic analyses indicate first that thousands of distinct transmission chains are co-circulating among both heterosexuals and MSM in the Netherlands. Second, we use these data to estimate the size distribution of complete transmission chains and find higher levels of in-country transmission, in more and larger transmission chains, than the directly observed phylogenetic data suggest. The estimated transmission chains amongst both risk groups infected with both B and non-B subtypes were highly heterogeneous in size, with most introductions showing no or very little onward transmission in the Netherlands. Most non-B onward transmission among heterosexuals was in transmission chains size 2–5, and among MSM in chains size at least 10. Correspondingly, we estimate four out of 10 heterosexual and six out of 10 MSM non-B infections in the Netherlands were acquired in-country, compared with almost eight of 10 heterosexual and MSM subtype B infections. There was little non-B transmission between risk groups in the Netherlands, whilst half of phylogenetically identified subtype B transmission chains amongst heterosexuals originated from Dutch MSM. The world regions of origin attributed to viral non-B introductions among both heterosexuals and MSM in the Netherlands originated mostly from sub-Saharan Africa and South and Southeast Asia and Western Europe. Subtype B introductions originated mostly from Western Europe, North and Latin America and the Caribbean.

Our findings are compatible with phylogenetic studies from other European countries, which provided little evidence of large domestic non-B HIV-1 sub-epidemics for heterosexuals, but several larger transmission chains amongst MSM [2,3,6,23]. However, our estimated proportions of in-country HIV acquisitions were consistently lower in phylogenetic analyses that did not adjust for incomplete sampling, showing that the observed phylogenetic subgraphs are challenging to interpret without further modelling that accounts for incomplete sequence sampling. The adjusted phylogenetically derived estimates of in-country HIV acquisition were broadly congruent with overall self-reported data concerning the most likely geographical origin of infection. For non-B-infected heterosexuals, the adjusted phylogenetically derived estimates of in-country HIV acquisition were higher than estimates from patients’ self-reported likely geographical origin of infection, but in line with survey estimates among heterosexual immigrants in a European survey, and in particular with survey estimates among heterosexual immigrants from sub-Saharan Africa [7]. The latter is particularly relevant, as the majority of our non-B heterosexual sample population was born in sub-Saharan Africa.

In comparison, among heterosexually infected individuals with subtype B, we find a much higher proportion of in-country acquisitions, primarily because half of transmission chains among subtype B heterosexuals were estimated to have originated in-country from MSM. Similar to what we see for non-B, these subsequently have not resulted in any major sub-epidemics amongst heterosexuals. The large subtype B subgraphs identified with predominantly heterosexual transmission include a high percentage of people from Curaçao and Suriname [24] (Supplementary Table S6). Consistent with the European aMASE survey, immigrants from Latin America and the Caribbean reported highest (71%) postmigration HIV acquisition [7].

Our study has several limitations. First, our phylogenetic analysis rests on the assumption that sufficient HIV sequences were publicly available at LANL to place the Dutch viral sequences into the correct epidemiologic context. Although only one ancestor separating distinct Dutch transmission chains needs to be in the background data set, it is certainly possible that some of the larger phylogenetically identified transmission chains in fact correspond to several smaller transmission chains. Our results may therefore overestimate the number of infections acquired in the Netherlands. Second, less than half of ATHENA participants had a viral sequence available for analysis, and the sampling fraction was higher among MSM compared to heterosexuals (44 versus 39%). Although we employed statistical models to account for incomplete sampling, the models have limitations. We assumed individuals are sampled at random with the same sampling probability in each risk group, and we cannot exclude the possibility that violation of these assumptions could introduce bias. Third, viral phylogenetic lineages were attributed the location of individuals at time of sampling, and this approach did not account for individual-level mobility. Several infections per chain could have occurred abroad amongst travellers and/or immigrants, or infections might have occurred in the Netherlands from short-term visitors. This could partly explain a respectively higher and lower proportion of in-country infections estimated by the phylogenetic approach compared to self-reported data. Alternatively, self-reported data on the likely origin of infection could be subject to reporting biases [25]. Finally, we have not estimated time trends, and it is possible that dynamics changed over time, in particular with recent test-and-treat policy and substantial reductions in new diagnose [26].

This study shows HIV-1 non-B subtypes are spreading in the Netherlands in many distinct transmission chains, which are significantly smaller in size than transmission chains of subtype B, both for heterosexuals and for MSM. Nevertheless, we estimate that a substantial proportion of non-B infections among heterosexuals and MSM are acquired within the Netherlands, and find that the discrepancy between previous phylogenetic studies of in-country transmission and recent survey estimates into the proportion of in-country transmission could be explained by incomplete sequence sampling of infected individuals.

## Acknowledgements

The members of the ATHENA observational cohort are as follows. Asterisks denote site coordinating physicians.

**Amsterdam UMC, locatie AMC, Amsterdam:***HIV treating physicians:* M. van der Valk^∗^, S.E. Geerlings, A. Goorhuis, J.W. Hovius, B. Lempkes, F.J.B. Nellen, T. van der Poll, J.M. Prins, P. Reiss, V. Spoorenberg, M. van Vugt, W.J. Wiersinga, F.W.M.N. Wit. *HIV nurse consultants:* M. van Duinen, J. van Eden, A.M.H. van Hes, F.J.J. Pijnappel, S.Y. Smalhout, A.M. Weijsenfeld. *HIV clinical virologists/chemists:* S. Jurriaans, N.K.T. Back, H.L. Zaaijer, B. Berkhout, M.T.E. Cornelissen, C.J. Schinkel, K.C. Wolthers.

**Amsterdam UMC, locatie VUmc, Amsterdam:***HIV treating physicians:* E.J.G. Peters^∗^, M.A. van Agtmael, R.S. Autar, M. Bomers, K.C.E. Sigaloff. *HIV nurse consultants:* M. Heitmuller, L.M. Laan. *HIV clinical virologists/chemists:* R. van Houdt, M. Jonges.

**Emma Kinderziekenhuis (Amsterdam UMC, locatie AMC):***HIV treating physicians:* M. van der Kuip, D. Pajkrt. *HIV nurse consultants:* C. de Boer, A.M. Weijsenfeld.

**Admiraal De Ruyter Ziekenhuis, Goes:***HIV treating physicians:* M. van den Berge^∗^, A. Stegeman. *HIV nurse consultants:* S. Baas, L. Hage de Looff. *HIV clinical virologists/chemists:* A. Reuwer, J. Veenemans, B. Wintermans.

**Catharina Ziekenhuis, Eindhoven:***HIV treating physicians:* M.J.H. Pronk^∗^, H.S.M. Ammerlaan. *HIV nurse consultants:* D.N.J. van den Bersselaar, E.S. de Munnik. *HIV clinical virologists/chemists:* B. Deiman, A.R. Jansz, V. Scharnhorst, J. Tjhie, M.C.A. Wegdam.

**DC Klinieken Lairesse - Hiv Focus Centrum:***HIV treating physicians: M. van der Valk*^∗^, A. van Eeden, E. Hoorenborg, J. Nellen,. *HIV nurse consultants:* W. Brokking, L.J.M. Elsenburg, H. Nobel. *HIV clinical virologists/chemists:* C.J. Schinkel.

**ETZ (Elisabeth-TweeSteden Ziekenhuis), Tilburg:***HIV treating physicians:* M.E.E. van Kasteren^∗^, M.A.H. Berrevoets, A.E. Brouwer. *HIV nurse consultants:* A. Adams, R. van Erve, B.A.F.M. de Kruijf-van de Wiel, S. Keelan-Phaf, B. van de Ven. *HIV data collection:* B.A.F.M. de Kruijf-van de Wiel, *HIV clinical virologists/chemists:* A.G.M. Buiting, J.L. Murck.

**Erasmus MC, Rotterdam:***HIV treating physicians:* T.E.M.S. de Vries-Sluijs^∗^, H.I. Bax, E.C.M. van Gorp, M. de Mendonça Melo, E. van Nood, J.L. Nouwen, B.J.A. Rijnders, C. Rokx, C.A.M. Schurink, L. Slobbe, A. Verbon. *HIV nurse consultants:* N. Bassant, J.E.A. van Beek, M. Vriesde, L.M. van Zonneveld. *HIV data collection:* J. de Groot. *HIV clinical virologists/chemists:* C.A.B. Boucher, M.P.G Koopmans, J.J.A van Kampen.

**Erasmus MC–Sophia, Rotterdam:***HIV treating physicians:* P.L.A. Fraaij, A.M.C. van Rossum, C.L. Vermont. *HIV nurse consultants:* L.C. van der Knaap, E. Visser.

**Flevoziekenhuis, Almere:***HIV treating physicians:* J. Branger^∗^, R.A. Douma. *HIV nurse consultant:* A.S. Cents-Bosma, C.J.H.M. Duijf-van de Ven.

**HagaZiekenhuis, Den Haag:***HIV treating physicians:* E.F. Schippers^∗^, C. van Nieuwkoop. *HIV nurse consultants:* J. Geilings, S. van Winden. *HIV data collection:* G. van der Hut. *Klinische viroloog/chemicus:* N.D. van Burgel.

**HMC (Haaglanden Medisch Centrum), Den Haag:***HIV treating physicians:* E.M.S. Leyten^∗^, L.B.S. Gelinck, F. Mollema. *HIV nurse consultants:* S. Davids-Veldhuis, C. Tearno, G.S. Wildenbeest. *HIV clinical virologists/chemists:* E. Heikens.

**Isala, Zwolle:***HIV treating physicians:* P.H.P. Groeneveld^∗^, J.W. Bouwhuis, A.J.J. Lammers. *HIV nurse consultants:* S. Kraan, A.G.W. van Hulzen, M.S.M. Kruiper. *HIV data collection:* G.L. van der Bliek, P.C.J. Bor. *HIV clinical virologists/chemists:* S.B. Debast, G.H.J. Wagenvoort.

**Leids Universitair Medisch Centrum, Leiden:***HIV treating physicians:* A.H.E. Roukens ^∗^, M.G.J. de Boer, H. Jolink, M.M.C. Lambregts, A.H.E. Roukens, H. Scheper. *HIV nurse consultants:* W. Dorama, N. van Holten. *HIV clinical virologists/chemists:* E.C.J. Claas, E. Wessels.

**Maasstad Ziekenhuis, Rotterdam:***HIV treating physicians:* J.G. den Hollander^∗^, R. El Moussaoui, K. Pogany. *HIV nurse consultants:* C.J. Brouwer, J.V. Smit, D. Struik-Kalkman. *HIV data collection:* T. van Niekerk. *HIV clinical virologists/chemists:* O. Pontesilli, C. van Tienen.

**Maastricht UMC+, Maastricht:***HIV treating physicians:* S.H. Lowe^∗^, A.M.L. Oude Lashof, D. Posthouwer, M.E. van Wolfswinkel. *HIV nurse consultants:* R.P. Ackens, K. Burgers, J. Schippers. *HIV data collection:* B. Weijenberg-Maes. *HIV clinical virologists/chemists:* J.J.M. Coremans, I.H.M. van Loo.

**Medisch Centrum Leeuwarden, Leeuwarden:***HIV treating physicians:* M.G.A. van Vonderen^∗^, L.M. Kampschreur. *HIV nurse consultants:* S. Faber, R. Steeman-Bouma. *HIV clinical virologists/chemists:* A. Al Moujahid.

**Medisch Spectrum Twente, Enschede:***HIV treating physicians:* G.J. Kootstra^∗^, C.E. Delsing. *HIV nurse consultants:* M. van der Burg-van de Plas, L. Scheiberlich.

**Noordwest Ziekenhuisgroep, Alkmaar:***HIV treating physicians:* W. Kortmann^∗^, G. van Twillert^∗^, R. Renckens, J. Wagenaar. *HIV nurse consultants: & HIV data collection:* D. Ruiter-Pronk, F.A. van Truijen-Oud. *HIV clinical virologists/chemists:* J.W.T. Cohen Stuart, ER. Jansen, M. Hoogewerf, W. Rozemeijer, W. A. van der Reijden, J.C. Sinnige.

**OLVG, Amsterdam:***HIV treating physicians:* K. Brinkman^∗^, G.E.L. van den Berk, W.L. Blok, K.D. Lettinga, M. de Regt, W.E.M. Schouten, J.E. Stalenhoef, J. Veenstra, S.M.E. Vrouenraets. *HIV nurse consultants:* H. Blaauw, G.F. Geerders, M.J. Kleene, M. Kok, M. Knapen, I.B. van der Meché, E. Mulder-Seeleman, A.J.M. Toonen, S. Wijnands, E. Wttewaal. *HIV clinical virologists:* T.J.W. van de Laar, D. Kwa.

**Radboudumc, Nijmegen:***HIV treating physicians:* R. van Crevel^∗^, K. van Aerde, A.S.M. Dofferhoff, S.S.V. Henriet, H.J.M. ter Hofstede, J. Hoogerwerf, M. Keuter, O. Richel. *HIV nurse consultants:* M. Albers, K.J.T. Grintjes-Huisman, M. de Haan, M. Marneef. *HIV clinical virologists/chemists:* J. Rahamat-Langendoen, F.F. Stelma. *Klinisch farmacoloog:* D. Burger.

**Rijnstate, Arnhem:***HIV treating physicians:* E.H. Gisolf^∗^, R.J. Hassing, M. Claassen. *HIV nurse consultants:* G. ter Beest, P.H.M. van Bentum, N. Langebeek. *HIV clinical virologists/chemists:* C.M.A. Swanink, R. Tiemessen.

**Spaarne Gasthuis, Haarlem:***HIV treating physicians:* S.F.L. van Lelyveld^∗^, R. Soetekouw. *HIV nurse consultants:* L.M.M. van der Prijt, J. van der Swaluw. *HIV clinical virologists/chemists:* W.A. van der Reijden, R. Jansen, B.L. Herpers, D.Veenendaal.

**Medisch Centrum Jan van Goyen, Amsterdam:***HIV treating physicians* D.W.M. Verhagen, F.N. Lauw. *HIV nurse consultants:* M.C. van Broekhuizen.

**Universitair Medisch Centrum Groningen, Groningen:***HIV treating physicians:* W.F.W. Bierman^∗^, M. Bakker, J. Kleinnijenhuis, E. Kloeze, A. Middel, D.F. Postma, E.H. Schölvinck, Y. Stienstra, A.R. Verhage, M. Wouthuyzen-Bakker. *HIV nurse consultants:* A. Boonstra, H. de Groot-de Jonge, M.M.M. Maerman, P.A. van der Meulen, D.A. de Weerd. *HIV clinical virologists/chemists:* H.G.M. Niesters, C.C. van Leer-Buter, M. Knoester.

Universitair Medisch Centrum Groningen/Beatrix Kinderziekenhuis, Groningen:

*HIV treating physicians:* E.H. Schölvinck, A.R. Verhage. *HIV nurse consultants:* H. de Groot-de Jonge. *HIV clinical virologists/chemists:* H.G.M. Niesters, C.C. van Leer-Buter, M. Knoester.

**Universitair Medisch Centrum Utrecht, Utrecht:***HIV treating physicians:* A.I.M. Hoepelman^∗^, R.E. Barth, A.H.W. Bruns, P.M. Ellerbroek, T. Mudrikova, J.J. Oosterheert, E.M. Schadd, B.J. van Welzen. *HIV nurse consultants:* K. Aarsman, B.M.G. Griffioen-van Santen, I. de Kroon. *HIV data collection:* M. van Berkel, C.S.A.M. van Rooijen. *HIV clinical virologists/chemists:* L.M. Hofstra, R. Schuurman, A.M.J. Wensing.

**Wilhelmina Kinderziekenhuis, UMC Utrecht, Utrecht:***HIV treating physicians:* L.J. Bont, S.P.M. Geelen, Y.G.T. Loeffen, T.F.W. Wolfs. *HIV nurse consultants:* N. Nauta.

**Sint Elisabeth Hospitaal, Willemstad, Curaçao:***HIV treating physicians:* E.O.W. Rooijakkers, D. van de Wetering. *HIV nurse consultants*: A. Alberto. *Data collection:* I. van der Meer. *HIV clinical virologists/ chemists:* A. Rosingh, T. Halaby

**Coordinating centre**

*Director:* P. Reiss

Deputy-director: S. Zaheri

*HIV data analysis:* A.C. Boyd, D.O. Bezemer, A.I. van Sighem, C. Smit, F.W.M.N. Wit

Data HIV data management and quality control: M.M.J. Hillebregt, T.J. Woudstra, T. Rutkens

HIV data monitoring: D. Bergsma, L. van de Sande, S. van der Vliet, A. Scheijgrond, K.J. Lelivelt

*HIV data collection:* L.G.M. de Groot-Berndsen, M. van den Akker, Y. Bakker, A. El Berkaoui, M. Bezemer, N.M. Brétin, E.A. Djoechro, M. Groters, C.R.E. Lodewijk, E.G.A. Lucas, L. Munjishvili, F. Paling, B.M. Peeck, C.M.J. Ree, R. Regtop, YM.C. Ruijs-Tiggelman, M.J.C. Schoorl, P.P. Schnörr, E.M Tuijn, D.P. Veenenberg, K.M. Visser, E.C.M Witte, R.J. Loenen. Patiëntregistratie: Y.M.C. Ruijs-Tiggelman, D. Bergsma.

Author contributions: Conceived and designed the study: DB AB MH CF OR. Performed the analyses: DB AB MH OR. Patient enrolment and data collection: AvS MC EW JvK TvdL PR. Wrote the first draft of the manuscript: DB OR. Contributed to the writing of the manuscript: AB MH AvS CF MC EW JvK P TvL PR. Agree with the manuscript's results and conclusions: DB AB OR MH AvS TvdL PR CF MC EW JvK. All authors have read, and confirm that they meet, ICMJE criteria for authorship.

### Conflicts of interest

None.

## Supplementary Material

Supplemental Digital Content

## Supplementary Material

Supplemental Digital Content

## Supplementary Material

Supplemental Digital Content

## Supplementary Material

Supplemental Digital Content

## Supplementary Material

Supplemental Digital Content

## Supplementary Material

Supplemental Digital Content

## Supplementary Material

Supplemental Digital Content

## Supplementary Material

Supplemental Digital Content

## Supplementary Material

Supplemental Digital Content
