# Supplementary material for: Many but small HIV-1 non-B transmission chains in the Netherlands
Source: AIDS. 2021 Oct 5;36(1):83–94. doi: 10.1097/QAD.0000000000003074 (PMC8655833; doi:10.1097/QAD.0000000000003074)
Supplement: Supplemental Digital Content [file aids-36-083-s001.pdf]

In-country acquisition of HIV-1 non-B infection within the Netherlands is frequent, but results in limited onward transmission

Supplementary Text

Methodological details and additional results

Bezemer et al.

Section 1: phyloscanner command line options

```
Phyloscanner_analyse_trees.R infile outfileString s,0 -m 1e-5 -x "[A-Z]*[0-9]*.*_samp([A-Z]*)_born[A-Z]*_inf[A-Z]*_.*" -v 1 -ow -rda
```

Section 2: Estimating the proportion of in-country HIV acquisitions.

This text presents the statistical framework for estimating the proportion of HIV acquisitions under a negative Binomial branching process model. The data used consists of the size distribution of phylogenetically likely transmission chains that circulate in a country of interest, here the Netherlands; numbers of sampled and unsampled infected individuals; and labels on the phylogenetically likely origin of the identified transmission chains.

Size distribution of complete transmission chains. We start by considering the probability p_j that a transmission chain has final size j under a Negative Binomial offspring distribution. Following [1]and [2],

$$p_j = \frac{\Gamma(\kappa j + j - 1)}{\Gamma(\kappa j)\Gamma(j + 1)} \frac{({R_0}/{\kappa})^{j-1}}{(1 + ({R_0}/{\kappa}))^{\kappa j + j - 1}}$$

where R_0 is the expected number of cases caused by an infected individual, and κ is the dispersion parameter of the Negative Binomial offspring distribution. The variance to mean ratio of the offspring distribution is $\phi = 1 + R_0/\kappa$, and so it is advantageous to re-express p_j in terms of R_0 and ϕ. To obtain parameters with positive support, we further consider $\upsilon = \phi - 1$. This gives

$$p_j = \frac{\Gamma(\frac{R_0}{\upsilon} j + j - 1)}{\Gamma(\frac{R_0}{\upsilon} j)\Gamma(j + 1)} \frac{\upsilon^{j-1}}{(1 + \upsilon)^{\kappa j + j - 1}}$$

Size distribution of partially sampled transmission chains. If we assume that infected individuals are sampled at random with probability ρ in any of the unobserved transmission chains, we have that the probability q_j that a partially sampled transmission chain has final size j is

$$q_j = \left[\sum_{k=j}^{\infty} p_k \, Binom(j; k, \rho)\right] \Big/ \left[1 - \sum_{k=1}^{\infty} p_k \, Binom(j; k, \rho)\right],$$

where the second term in square brackets accounts for the fact partially sampled transmission chains are at least of size 1 [3].

Likelihood of size distribution of partially sampled transmission chains. Consider the number x_j of partially observed transmission chains that are of size $j = 1, \dots J$, where J is the size of the largest observed transmission chains. Assuming that all transmission chains evolved independently over time, the log likelihood of the partially observed transmission chains under the Negative Binomial branching process model is

$$l(x \mid R_0, \upsilon, \rho) = \sum_{j=1}^{J} x_j \log q_j.$$

Bayesian model. To estimate the parameters of the branching process model, we considered the Bayesian model

$$p(R_0, \upsilon, \rho \mid x) \propto \exp\big(l(x \mid R_0, \upsilon, \rho)\big)\ p(R_0)\ p(\upsilon)\ p(\rho),$$

where the prior distribution of R_0 was specified as a $Beta(2,2)$ density, the prior distribution of υ was specified as an $Exp(1)$ density, and the prior on the sampling probability ρ was set to a $Beta(N^s + 0.5, N - N^s + 0.5)$ density, where N is the number of infected individuals in the cohort and N^s is the number of sampled individuals. The posterior distribution was estimated with Stan version 2.23 [4]. Run-times were on the order of 10-40 minutes on 2.3 Ghz Intel iCore9, depending on the size of J, to generate approximately 3,000 effective samples from the marginal posterior distributions. The infinite sum in q_j was capped at $10 \times J/(N^s/N)$, and choosing higher cut-off values did not change our results within 4 decimal digits.

Posterior predictive chain sizes. We were next interested in sampling sizes of the actual, unobserved transmission chains from the posterior predictive distribution

$$p(j^*|x) = \int p_{j^*}\ p(R_0, \upsilon, \rho|x) d(R_0, \upsilon, \rho).$$

Given samples from the posterior distribution $p(R_0, \upsilon, \rho;\ x)$, this is straightforward to achieve because the probability p_j of the size of the actual transmission chains is available in closed form. We simulated $k = 1,2, \dots, K^*$ actual transmission chains until the sum N^* of the size of the K^* transmission chains was equal to or for the first time larger than the number N of infected individuals. To be clear, note that the number of actual transmission chains K^* is a random variable, determined through the posterior predictive distribution of actual transmission chain sizes $p(j^*|x)$. The posterior predictive probability of the proportion of infections that correspond to a viral introduction, γ,was then calculated as

$$\gamma = K^*/N^*,$$

because each distinct transmission chain has one index case. This process was repeated 1,000 times to obtain Monte Carlo estimates of the posterior predictive median and posterior predictive 95% credibility intervals.

Proportion of in-country HIV acquisitions. To estimate the proportion of in-country HIV-acquisition, we had to account for the fact that a subset of the predicted transmission chains was introduced by individuals from the Netherlands who are in a different risk group (e.g. MSM when considering transmission chains among heterosexual individuals). Based on the phylogenetic analysis, we obtained the number of phylogenetically likely transmission chains n_r with origin in world region r. We assumed that the origin label r=1,…,R of each chain was independently drawn at random from a Categorical distribution with probability π_r, where

$\sum_{r=1}^{R} \pi_r = 1$. We further assumed a Dirichlet prior distribution on the origin probabilities π_r with hyper-parameter 1. Based on the phylogenetic data, the posterior distribution of the origin probabilities π_r thus followed a Dirichlet distribution with hyperparameters $n_r + 1$. Sampling from this distribution allowed us to assign origin locations to the K^* actual transmission chains that were drawn according to the posterior predictive distribution of actual transmission chain sizes. Denote by E^* the number of posterior predicted transmission chains K^* that had their origin assigned to outside the Netherlands. Thus, the proportion of in-country HIV acquisitions was estimated by

$$\alpha = \frac{N^* - E^*}{N^*}$$

In total 1,000 Monte Carlo samples were drawn to quantify the posterior predictive median and posterior predictive 95% credibility intervals of α.

References

1 Nishiura H, Yan P, Sleeman CK, Mode CJ. Estimating the transmission potential of supercritical processes based on the final size distribution of minor outbreaks. *Journal of Theoretical Biology* 2012; **294**:48–55.

2 Blumberg S, Lloyd-Smith JO. Inference of R0 and Transmission Heterogeneity from the Size Distribution of Stuttering Chains. *PLoS Computational Biology* 2013; **9**:1–17.

3 Blumberg S, Lloyd-Smith JO. Comparing methods for estimating R0from the size distribution of subcritical transmission chains. *Epidemics* 2013; **5**:131–145.

4 Carpenter B, Gelman A, Hoffman MD, Lee D, Goodrich B, Betancourt M, *et al.* Stan: A probabilistic programming language. *Journal of Statistical Software* 2017; **76**. doi:10.18637/jss.v076.i01
