# Supplementary material for: Many but small HIV-1 non-B transmission chains in the Netherlands
Source: AIDS. 2021 Oct 5;36(1):83–94. doi: 10.1097/QAD.0000000000003074 (PMC8655833; doi:10.1097/QAD.0000000000003074)
Supplement: Supplemental Digital Content [file aids-36-083-s003.pdf]

Table S2. Demographics of the study population, HIV-1 infected participants of the ATHENA study, the Netherlands.

	subtype A1	CRF01AE	CRF02AG	CRF06-cpx	subtype C	subtype D	subtype F1	subtype G	Total non-B	subtype B	Other, not in study	Total in study	All patients in ATHENA
**Number of sequences**	417	338	770	72	615	111	93	173	2,589	8,382	266	10,971	26,881
**Sampling year median**	2009	2010	2009	2009	2009	2007	2012	2009	2009	2008	2010	2008	-
IQR	2006-2013	2006-2014	2006-2012	2007-2013	2005-2011	2004-2012	2009-2014	2005--2012	2005-2012	2005-2011	2006-2014	2005-2012	
**MSM % (n)**	19 (78)	43 (146)	18 (136)	8 (6)	14 (86)	13 (14)	56 (52)	10 (18)	21 (536)	77 (6,413)	28 (75)	63 (6,949)	59 (15,882)
**HSX (Heterosexual) % (n)**	61 (255)	46 (157)	64 (496)	72 (52)	66 (404)	64 (71)	33 (31)	71 (122)	61 (1,588)	16 (1,344)	53 (141)	27 (2,932)	28 (7,608)
Female	62 (157)	42 (66)	58 (289)	58 (30)	60 (241)	70 (50)	58 (18)	55 (67)	58 (918)	46 (617)	58 (82)	52 (1,535)	53 (4,040)
Male	38 (98)	58 (91)	42 (207)	42 (22)	40 (163)	30 (21)	42 (13)	45 (55)	42 (670)	54 (727)	42 (59)	48 (1,397)	47 (3,568)
**Reported Infection region MSM**	% n	% n	% n	% n	% n	% n	% n	% n	% n	% n	% n	% n	% n
NL	59 46	39 57	61 83	33 2	53 46	57 8	73 38	39 7	54 287	70 4,459	55 41	68 4,746	64 10,174
Elsewhere	15 12	25 36	10 14	67 4	14 12	29 4	8 4	17 3	17 89	8 523	19 14	9 612	13 2,109
Unknown	26 20	36 53	29 39	0 0	33 28	14 2	19 10	44 8	30 160	22 1,431	27 20	23 1,591	23 3,599
**Reported infection region HSX**	% n	% n	% n	% n	% n	% n	% n	% n	% n	% n	% n	% n	% n
NL	22 57	18 28	22 108	21 11	17 68	25 18	39 12	20 25	21 327	59 791	18 26	38 1,118	35 2,647
Elsewhere	47 119	51 80	41 202	44 23	51 208	59 42	35 11	42 51	46 736	16 214	45 63	32 950	39 2,954
Unknown	31 79	31 49	38 186	35 18	32 128	15 11	26 8	38 46	33 525	25 339	37 52	29 864	26 2,007
**Region origin MSM**	% n	% n	% n	% n	% n	% n	% n	% n	% n	% n	% n	% n	% n
Europe - Central	1 1	1 2	2 3	0 0	2 2	0 0	6 3	0 0	2 11	2 134	1 1	2 145	2 354
Europe - West	13 10	5 8	5 7	17 1	5 4	7 1	6 3	17 3	7 37	7 476	5 4	7 513	8 1,230
Europe – East and Central Asia	5 4	1 1	1 2	0 0	6 5	0 0	0 0	6 1	1 8	1 37	0 0	1 45	1 116
Latin America and the Caribbean	5 4	5 7	6 8	0 0	0 0	7 1	8 4	0 0	5 29	5 323	8 6	5 352	6 906
Netherlands	54 42	57 83	63 86	33 2	67 58	50 7	75 39	56 10	61 327	70 4,513	49 37	70 4,840	68 10,861
North Africa and Middle East	5 4	4 6	1 2	0 0	3 3	14 2	2 1	6 1	4 19	2 125	12 9	2 144	2 359
North America	1 1	1 1	1 1	0 0	0 0	0 0	0 0	0 0	1 3	2 119	4 3	2 122	2 316
Southeast Asia and Oceania	4 3	22 32	1 2	0 0	1 1	0 0	2 1	17 3	8 42	3 188	5 4	3 230	4 572
Suriname and Curaçao	5 4	3 4	8 11	0 0	2 2	0 0	2 1	0 0	4 22	7 424	9 7	6 446	6 876
sub-Saharan Africa	5 4	1 1	9 12	50 3	13 11	21 3	0 0	0 0	6 34	1 54	4 3	1 88	1 220
Unknown	1 1	1 1	1 2	0 0	0 0	0 0	0 0	0 0	1 4	0 20	1 1	0 24	0 72
**Region origin HSX**	% n	% n	% n	% n	% n	% n	% n	% n	% n	% n	% n	% n	% n
Europe - Central	2 4	0 0	1 3	6 3	0 1	0 0	10 3	1 1	1 15	1 16	1 2	1 31	2 118
Europe - West	3 7	4 6	1 3	0 0	1 2	1 1	3 1	5 6	2 26	4 49	2 3	3 75	3 253
Europe – East and Central Asia	6 15	0 0	0 0	0 0	0 0	1 1	0 0	0 0	1 16	1 7	0 0	1 23	1 48
Latin America and the Caribbean	0 1	0 0	1 6	2 1	0 1	1 1	10 3	1 1	1 14	4 52	3 4	2 66	3 216
Netherlands	17 44	52 81	15 73	4 2	20 81	20 14	26 8	16 20	20 323	52 693	16 22	35 1,016	35 2625
North Africa and Middle East	9 24	2 3	4 18	0 0	9 36	13 9	3 1	4 5	6 96	6 87	4 5	6 183	6 437
North America	0 0	1 1	0 0	0 0	0 1	0 0	0 0	0 0	0 2	0 4	1 1	0 6	0 13
Southeast Asia and Oceania	1 3	38 59	0 1	2 1	1 4	1 1	0 0	0 0	4 69	2 23	3 4	3 92	4 303
Suriname and Curaçao	1 3	2 3	3 14	6 3	2 8	1 1	10 3	6 7	3 42	28 377	1 1	14 419	12 946
sub-Saharan Africa	60 152	3 4	76 378	81 42	67 269	61 43	35 11	67 82	62 981	2 30	70 99	34 1,011	35 2625
Unknown	1 2	0 0	0 0	0 0	0 1	0 0	3 1	0 0	0 4	0 6	0 0	0 10	0 24
