# Supplementary material for: Many but small HIV-1 non-B transmission chains in the Netherlands
Source: AIDS. 2021 Oct 5;36(1):83–94. doi: 10.1097/QAD.0000000000003074 (PMC8655833; doi:10.1097/QAD.0000000000003074)
Supplement: Supplemental Digital Content [file aids-36-083-s004.pdf]

Table S2. Demographics of the international background sequences by subtype.

	**subtype A1**		**CRF01AE**		**CRF02AG**		**CRF06-cpx**		**subtype C**		**subtype D**		**subtype F1**		**subtype G**		**Total non-B**		**subtype B**		**Total**	
**Number of sequences**	7,339		20,275		5,713		792		25,446		3,367		1,699		1,550		66,181		73,677		139,859	
**Ssampling year median** (IQR)	2010 (2007-2014)		2012 (2009-2014)		2009 (2006-2014)		2009 (2007-2010)		2010 (2007-2013)		2009 (2007-2014)		2007 (2004-2012)		2008 (2006-2011)		2010 (2007-2013)		2009 (2005-2012)		2009 (2006-2013)	
**Sample Region**	**%**	**n**	**%**	**n**	**%**	**n**	**%**	**n**	**%**	**n**	**%**	**n**	**%**	**n**	**%**	**n**	**%**	**n**	**%**	**n**	**%**	**n**
Europe - Central	1	81	0	50	1	61	45	360	0	117	1	46	44	748	2	38	2	1,501	3	2,358	3	3,859
Europe - West	12	855	2	442	17	947	9	69	8	1,999	5	181	22	368	27	411	8	5,272	22	16,074	15	21,346
Europe – East and Central Asia	0	13	0	8	1	54	1	5	0	37	0	3	0	2	4	60	0	182	1	444	0	626
Latin America and the Caribbean	0	12	0	6	1	74	0	1	3	859	6	20	29	500	2	37	2	1,509	14	10,546	9	12,055
North Africa and Middle East	5	346	0	30	2	126	0	3	0	40	1	24	0	3	0	6	1	578	0	196	1	774
North America	3	245	1	157	5	272	1	10	3	852	2	72	1	16	4	66	3	1,690	42	30,732	23	32,422
South/East Asia and Oceania	2	112	96	19,493	3	181	3	24	10	2,581	1	25	1	13	7	109	34	22,538	18	12,963	25	35,501
Suriname and Curaçao	0	0	0	0	0	1	0	0	0	1	0	0	0	0	0	0	0	2	0	262	0	264
sub-Saharan Africa	77	5,675	0	89	70	3,997	40	320	75	18,960	89	2,996	3	49	53	823	50	32,909	0	102	24	33,012
