# Supplementary material for: Many but small HIV-1 non-B transmission chains in the Netherlands
Source: AIDS. 2021 Oct 5;36(1):83–94. doi: 10.1097/QAD.0000000000003074 (PMC8655833; doi:10.1097/QAD.0000000000003074)
Supplement: Supplemental Digital Content [file aids-36-083-s005.pdf]

Supplementary Table S4. Phylogenetically observed likely HIV-1 transmission chains amongst heterosexuals and MSM, results by subtype.

	Subtype	A1	CRF01AE	CRF02AG	CRF06-cpx	C	D	F1	G	Total non-B	B*
Analysis MSM	**Number of sequences**	78	146	136	6	86	14	52	18	536	6,413
	**Number of subtrees**	35	103	40	6	49	7	17	13	270	2,154
	Percentage of total sequences	45	71	29	100	57	50	33	72	50	34
	**Large subtrees size ≥10**	2	1	3	0	1	0	1	0	8	94
	Percentage of all subtrees	6	1	8	0	2	0	6	0	3	4
	**Number of sequences in subtrees size ≥10**	27	12	85	0	29	0	32	0	185	3,033
	Percentage of total sequences	35	8	63	0	34	0	62	0	35	47
Analysis heterosexuals	**Number of sequences**	255	157	496	52	404	71	31	122	1,588	1,344
	**Number of subtrees**	188	128	355	43	334	62	22	92	1,224	856
	Percentage of total sequences	74	82	72	83	83	87	71	75	77	63
	**Large subtrees size ≥10**	1	0	1	0	0	0	0	0	2	6
	Percentage of all subtrees	1	0	0	0	0	0	0	0	0	1
	**Number of sequences in subtrees size ≥10**	30	0	12	0	0	0	0	0	42	148
	Percentage of total sequences	12	0	2	0	0	0	0	0	3	11
