# Supplementary material for: Many but small HIV-1 non-B transmission chains in the Netherlands
Source: AIDS. 2021 Oct 5;36(1):83–94. doi: 10.1097/QAD.0000000000003074 (PMC8655833; doi:10.1097/QAD.0000000000003074)
Supplement: Supplemental Digital Content [file aids-36-083-s006.pdf]

Supplementary Table S5. Phylogeographic origin of observed likely transmission chains amongst heterosexuals and MSM, by subtype.

	subtype A1		CRF01AE		CRF02AG		CRF06-cpx		subtype C		subtype D		subtype F1		subtype G		Total non-B		subtype B	
**Origin of MSM subgraphs**	N	%	N	%	N	%	N	%	N	%	N	%	N	%	N	%	N	%	N	%
Europe - Central	0	0	1	1	0	0	0	0	0	0	0	0	3	18	0	0	4	1	44	2
Europe - West	18	51	1	1	10	25	1	17	6	12	0	0	9	53	2	15	47	17	723	34
Europe – East and Central Asia	0	0	0	0	0	0	0	0	0	0	0	0	0	0	3	23	3	1	6	0
Latin America and the Caribbean	1	3	0	0	0	0	0	0	4	8	0	0	2	12	0	0	7	3	198	9
Netherlands- heterosexuals	5	14	2	2	1	3	0	0	1	2	0	0	0	0	0	0	9	3	47	2
Netherlands – drug users	0	0	0	0	0	0	0	0	0	0	0	0	0	0	0	0	0	0	4	0
Netherlands – other/unknown	0	0	0	0	1	3	0	0	0	0	0	0	0	0	0	0	1	0	6	0
North Africa and Middle East	0	0	1	1	0	0	0	0	0	0	1	14	0	0	0	0	2	1	1	0
North America	0	0	0	0	0	0	0	0	0	0	0	0	0	0	0	0	0	0	615	29
Southeast Asia and Oceania	0	0	80	78	0	0	0	0	6	12	0	0	0	0	1	8	87	32	22	1
Suriname and Curaçao	0	0	0	0	0	0	0	0	0	0	0	0	0	0	0	0	0	0	8	0
sub-Saharan Africa	3	9	0	0	22	55	3	50	20	41	3	43	0	0	1	8	52	19	1	0
Unresolved	8	23	18	17	6	15	2	33	12	24	3	43	3	18	6	46	58	21	479	22
**Origin of heterosexual subgraphs**	N	%	N	%	N	%	N	%	N	%	N	%	N	%	N	%	N	%	N	%
Europe - Central	21	11	0	0	0	0	2	5	0	0	1	2	4	18	0	0	7	1	4	0
Europe - West	0	0	0	0	23	6	1	2	13	4	1	2	0	0	13	14	72	6	128	15
Europe – East and Central Asia	0	0	0	0	0	0	0	0	0	0	0	0	0	0	0	0	0	0	2	0
Latin America and the Caribbean	0	0	0	0	0	0	0	0	3	1	0	0	4	18	0	0	7	1	71	8
Netherlands – drug users	0	0	0	0	0	0	0	0	0	0	0	0	0	0	0	0	0	0	17	2
Netherlands- MSM	6	3	2	2	10	3	0	0	7	2	2	3	1	5	0	0	28	2	335	39
Netherlands – other/unknown	0	0	0	0	6	2	0	0	2	1	0	0	1	5	0	0	9	1	7	1
North Africa and Middle East	0	0	0	0	0	0	0	0	0	0	0	0	0	0	0	0	0	0	0	0
North America	0	0	0	0	2	1	0	0	2	1	0	0	0	0	0	0	4	0	87	10
Southeast Asia and Oceania	0	0	100	78	0	0	0	0	3	1	0	0	0	0	2	2	105	9	3	0
Suriname and Curaçao	0	0	0	0	0	0	0	0	0	0	0	0	0	0	0	0	0	0	21	2
sub-Saharan Africa	128	68	3	2	249	70	32	74	208	62	47	76	4	18	55	60	726	59	0	0
Unresolved	33	18	23	18	65	18	8	19	96	29	11	18	8	36	22	24	266	22	181	21
