# Supplementary material for: Many but small HIV-1 non-B transmission chains in the Netherlands
Source: AIDS. 2021 Oct 5;36(1):83–94. doi: 10.1097/QAD.0000000000003074 (PMC8655833; doi:10.1097/QAD.0000000000003074)
Supplement: Supplemental Digital Content [file aids-36-083-s008.pdf]

	subtype A1	CRF01AE	CRF02AG	CRF06-cpx	subtype C	subtype D	subtype F1	subtype G	total non-B		subtype B	
Size MSM	n	n	n	n	n	n	n	n	n	%	n	%
1	25	80	29	6	42	5	12	10	209	77.4	1486	68.99
2	4	17	4		4		4	1	34	12.6	286	13.28
3	2	2	3		1	1		2	11	4.1	110	5.11
4	1	2			1				4	1.5	63	2.92
5			1						1	0.4	39	1.81
6		1				1			2	0.7	27	1.25
7									0	0.0	22	1.02
8	1								1	0.4	14	0.65
9									0	0.0	13	0.60
10	1								1	0.4	9	0.42
11									0	0.0	7	0.32
12		1							1	0.4	6	0.28
13									0	0.0	4	0.19
14									0	0.0	5	0.23
15									0	0.0	3	0.14
16									0	0.0	4	0.19
17	1								1	0.4	2	0.09
18									0	0.0	6	0.28
19									0	0.0	3	0.14
20									0	0.0	1	0.05
21			1						1	0.4	5	0.23
22									0	0.0	2	0.09
23									0	0.0	1	0.05
24									0	0.0	1	0.05
25			1						1	0.4	1	0.05
27									0	0.0	2	0.09
29					1				1	0.4	0	0.0
32							1		1	0.4	0	0.0
33									0	0.0	1	0.05
34									0	0.0	1	0.05
35									0	0.0	2	0.09
36									0	0.0	3	0.14
37									0	0.0	2	0.09
39			1						1	0.4	0	0.0
40									0	0.0	1	0.05
43									0	0.0	1	0.05
44									0	0.0	1	0.05
45									0	0.0	1	0.05
46									0	0.0	2	0.09
47									0	0.0	1	0.05
50									0	0.0	1	0.05
52									0	0.0	2	0.09
53									0	0.0	1	0.05
55									0	0.0	1	0.05
63									0	0.0	1	0.05
64									0	0.0	1	0.05
67									0	0.0	1	0.05
75									0	0.0	1	0.05
78									0	0.0	1	0.05
81									0	0.0	1	0.05
99									0	0.0	1	0.05
116									0	0.0	1	0.05
141									0	0.0	1	0.05
143									0	0.0	1	0.05
250									0	0.0	1	0.05
	**subtype A1**	**CRF01AE**	**CRF02AG**	**CRF06-cpx**	**subtype C**	**subtype D**	**subtype F1**	**subtype G**	**total non-B**		**subtype B**	
Size HSX	n	n	n	n	n	n	n	n	n	%	n	%
1	165	110	274	35	280	54	16	71	1005	82.1	676	79.0
2	15	13	51	7	44	7	4	15	156	12.7	103	12.0
3	3	3	18	1	6	1	1	3	36	2.9	29	3.4
4	2	1	5		2		1	3	14	1.1	18	2.1
5			3		2				5	0.4	11	1.3
6	1		2						3	0.2	1	0.1
7	1		1						2	0.2	6	0.7
8		1							1	0.1	2	0.2
9									0	0.0	4	0.5
10									0	0.0	1	0.1
11									0	0.0	2	0.2
12			1						1	0.1	0	0.0
13									0	0.0	1	0.1
