# Supplementary material for: Many but small HIV-1 non-B transmission chains in the Netherlands
Source: AIDS. 2021 Oct 5;36(1):83–94. doi: 10.1097/QAD.0000000000003074 (PMC8655833; doi:10.1097/QAD.0000000000003074)
Supplement: Supplemental Digital Content [file aids-36-083-s009.pdf]

Supplementary Figures S1-S39. Reconstructed viral phylogenies of HIV-1 non-B subtypes A1, C, D, F, and G, circulating recombinant forms CRF01AE, CRF02AG, and CRF06-cpx, and subtype B. The viral phylogenies were reconstructed from partial HIV-1 pol sequences of participants enrolled in the Dutch national ATHENA cohort, and the corresponding collection of viral sequences from the Los Alamos National Lab HIV database. Viral phylogenetic lineages were associated to geographic regions based on the sampling location of sequenced individuals and transmission category in the Netherlands with maximum parsimony. External dots represent the sequenced samples. Colours of internal dots and branches represent the estimated ancestral location of the viral lineages. The subtype B tree analysis was divided in 31 different clades to make phyloscanner computationally feasible (S9-S39).

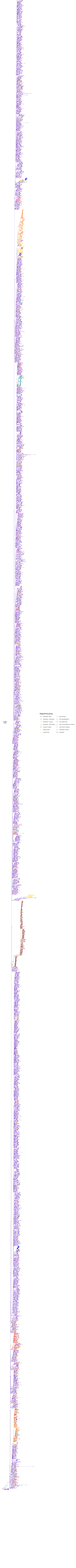
Region/risk group
Netherlands − MSM
Netherlands − heterosexual
Netherlands − drug user
Netherlands − other/unknown
Suriname / Curaçao
Western Europe
Central Europe
Eastern Europe
North Africa/Middle East
Sub−Saharan Africa
South− and Southeast Asia / Oceania
Latin America / Caribbean
United States / Canada
Unassigned

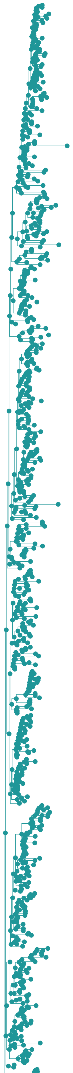

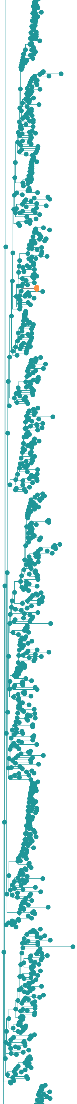

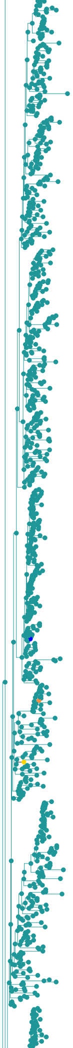

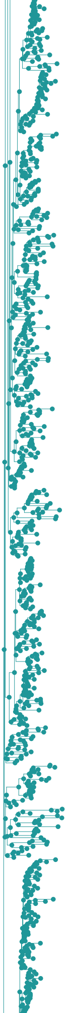

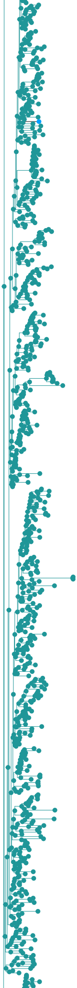

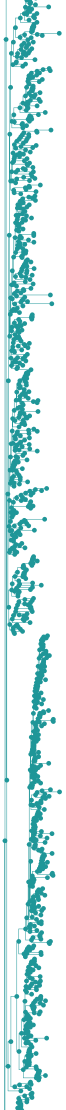

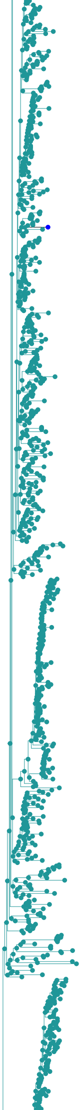

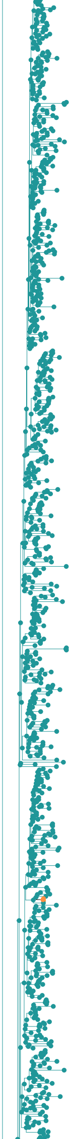

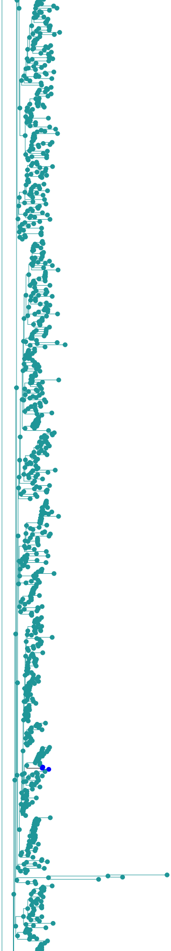

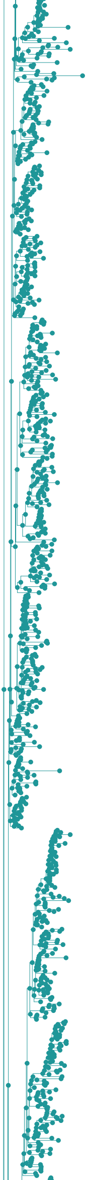

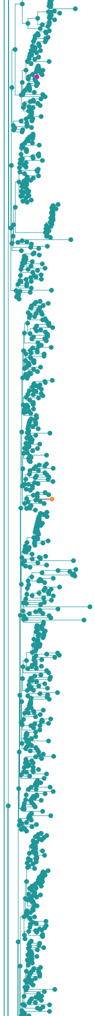

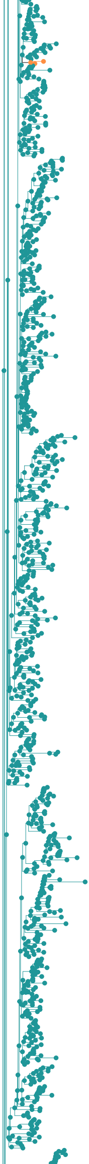

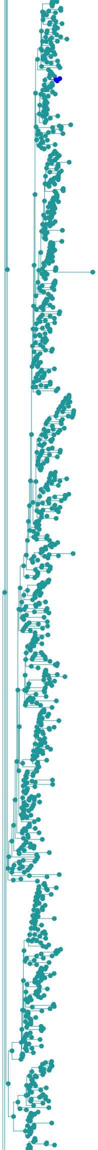

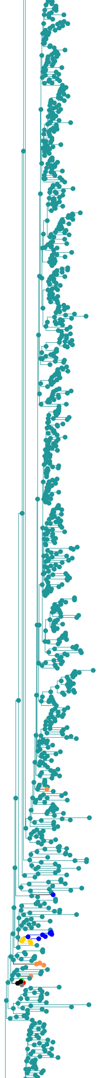

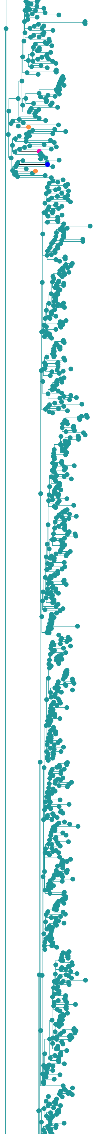

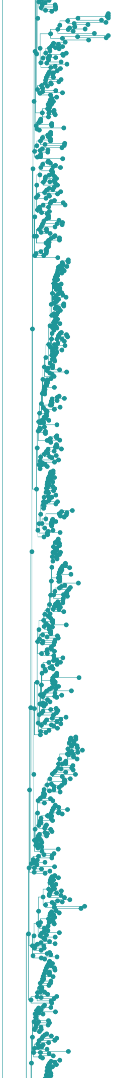

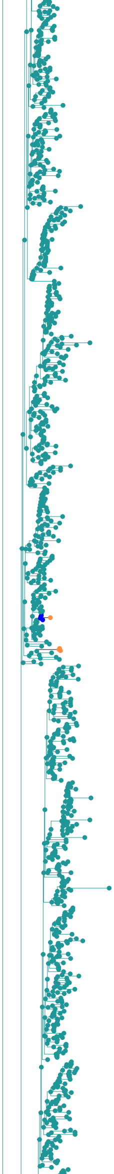

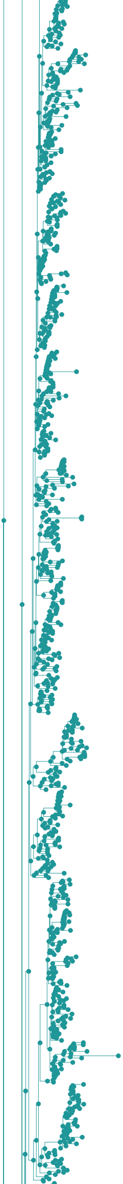

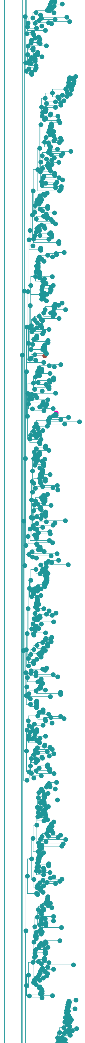

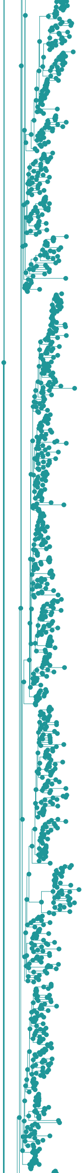

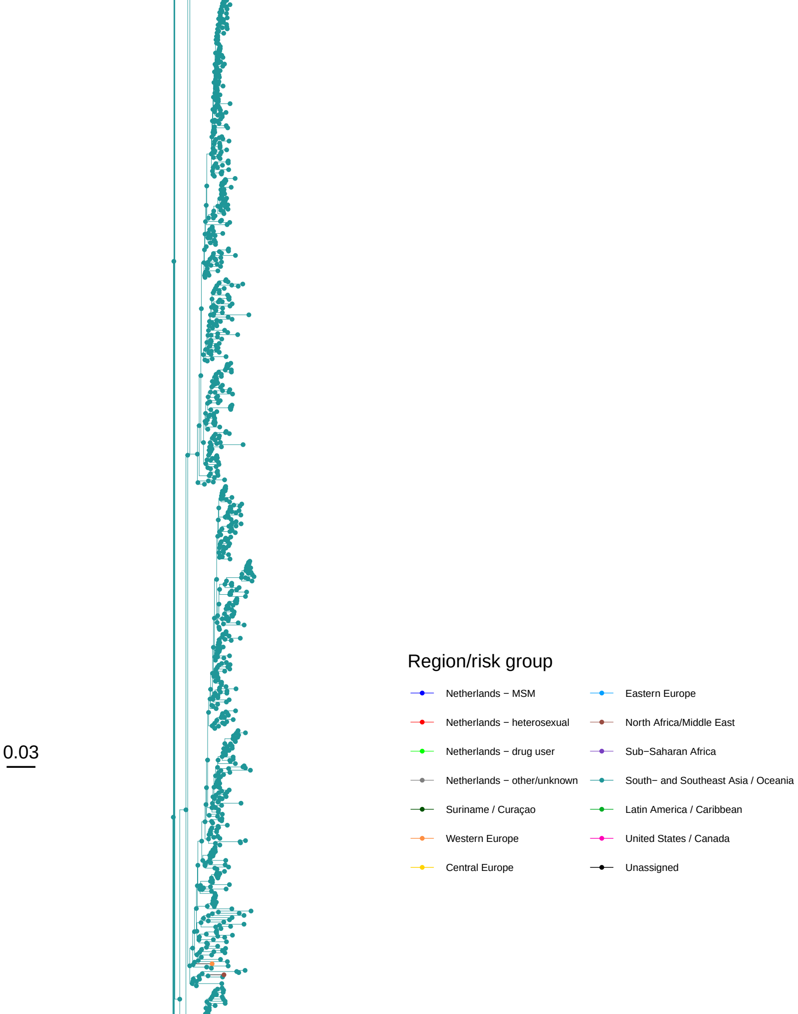
0.03
Region/risk group
Netherlands − MSM
Netherlands − heterosexual
Netherlands − drug user
Netherlands − other/unknown
Suriname / Curaçao
Western Europe
Central Europe
Eastern Europe
North Africa/Middle East
Sub−Saharan Africa
South− and Southeast Asia / Oceania
Latin America / Caribbean
United States / Canada
Unassigned

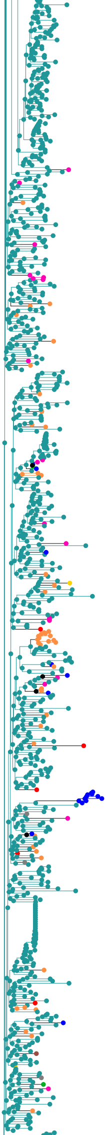

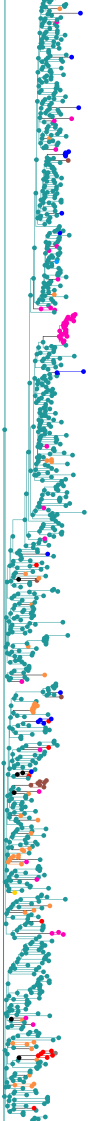

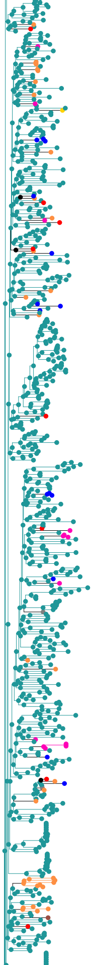

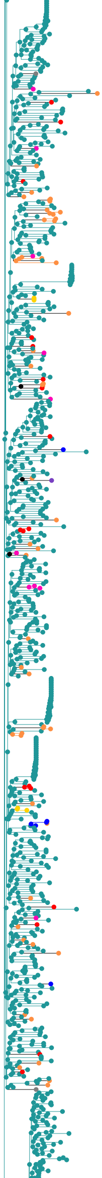

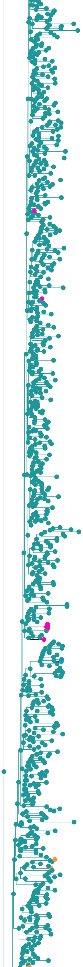

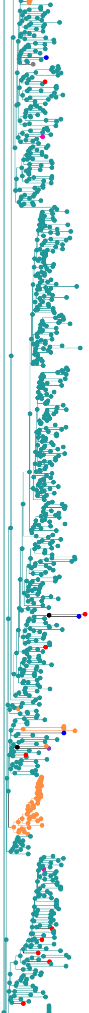

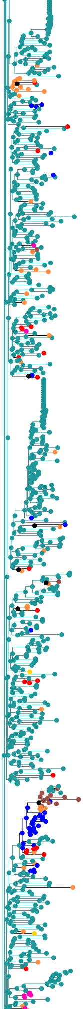

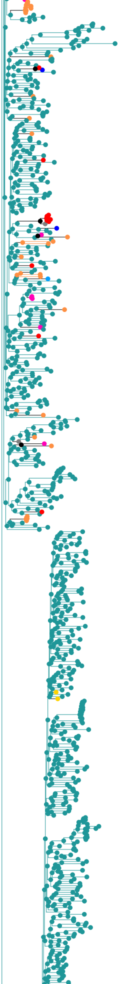

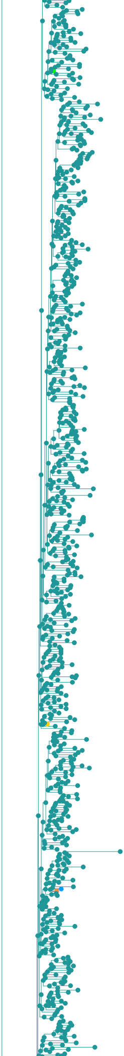

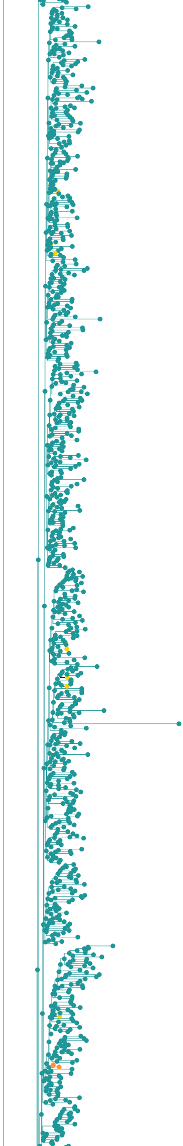

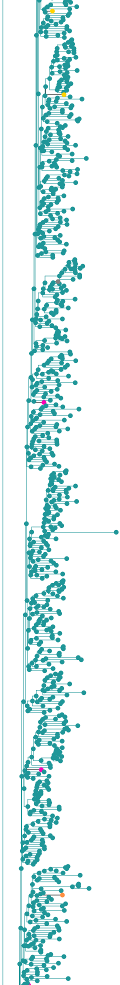

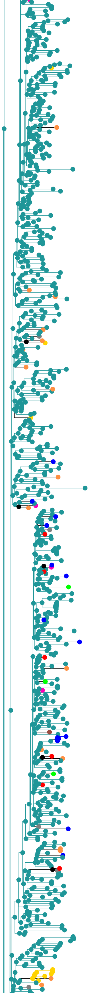

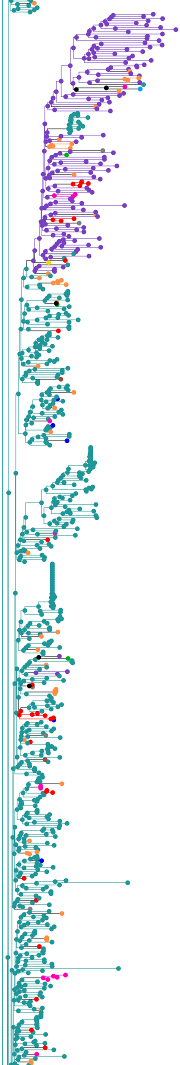

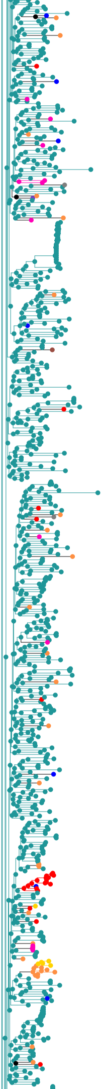

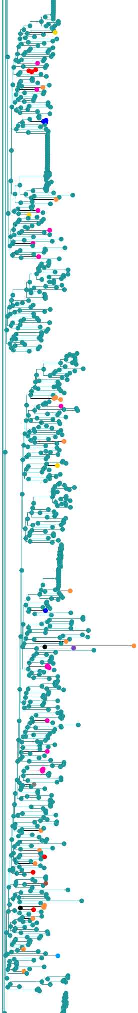

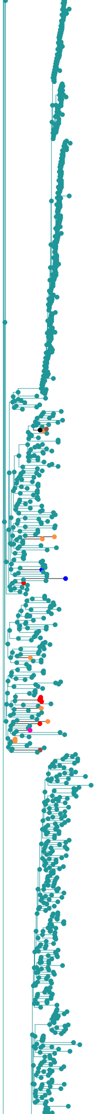

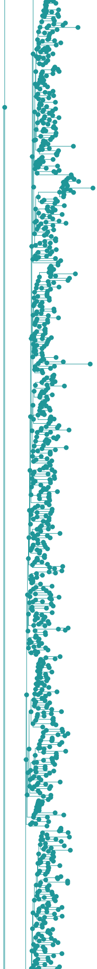

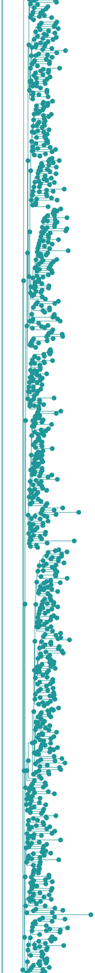

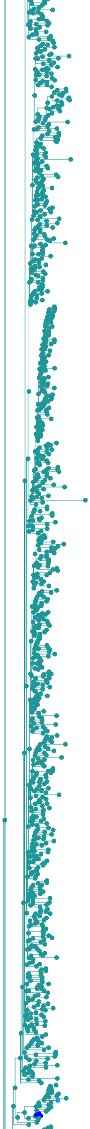

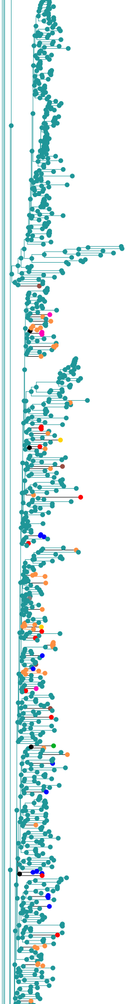

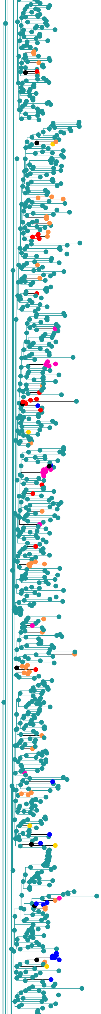

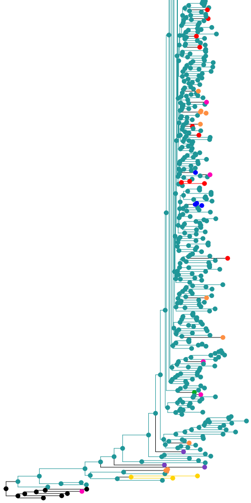

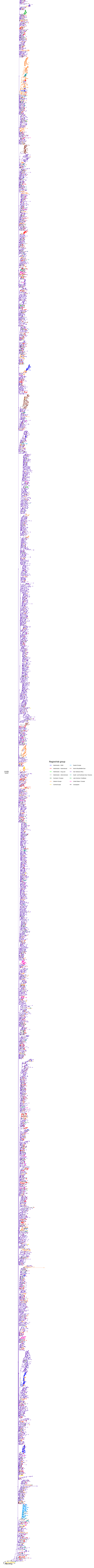
0.03
Region/risk group
Netherlands − MSM
Netherlands − heterosexual
Netherlands − drug user
Netherlands − other/unknown
Suriname / Curaçao
Western Europe
Central Europe
Eastern Europe
North Africa/Middle East
Sub−Saharan Africa
South− and Southeast Asia / Oceania
Latin America / Caribbean
United States / Canada
Unassigned

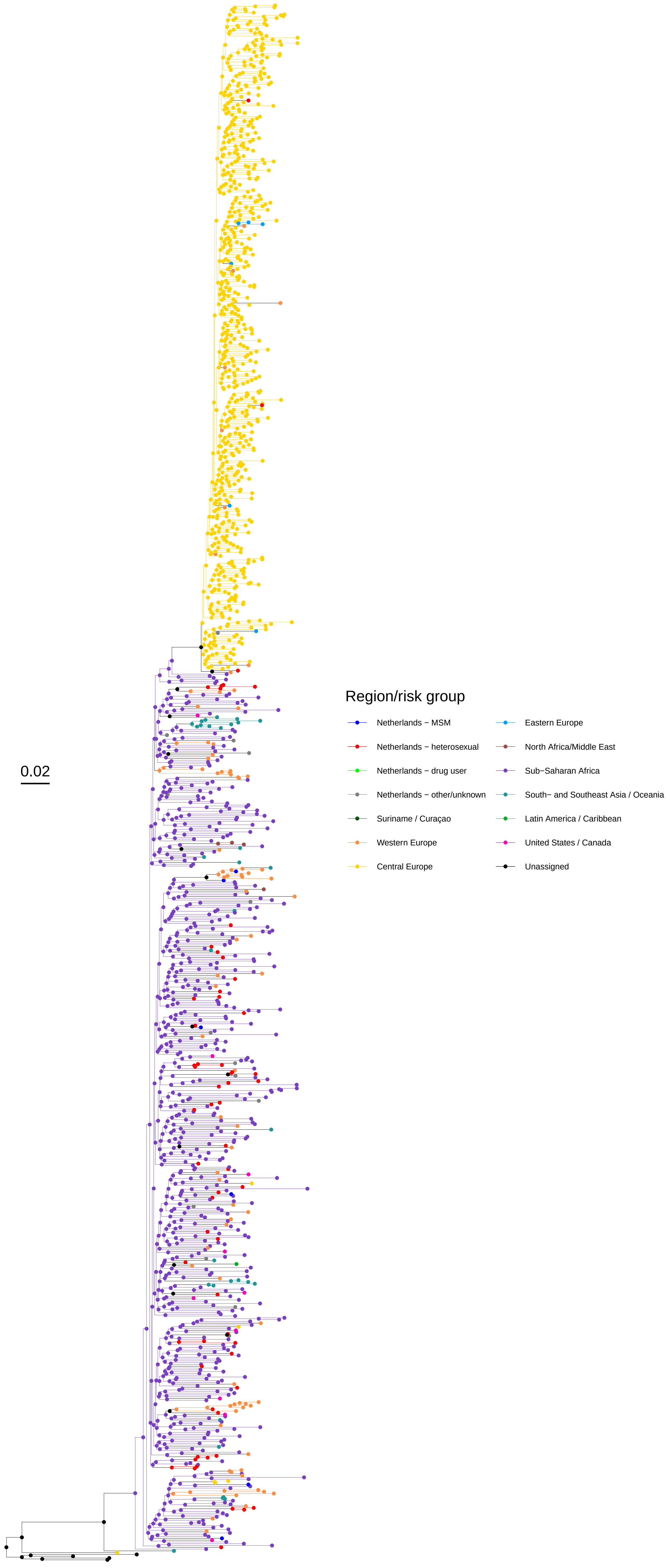
0.02
Region/risk group
Netherlands − MSM
Netherlands − heterosexual
Netherlands − drug user
Netherlands − other/unknown
Suriname / Curaçao
Western Europe
Central Europe
Eastern Europe
North Africa/Middle East
Sub−Saharan Africa
South− and Southeast Asia / Oceania
Latin America / Caribbean
United States / Canada
Unassigned

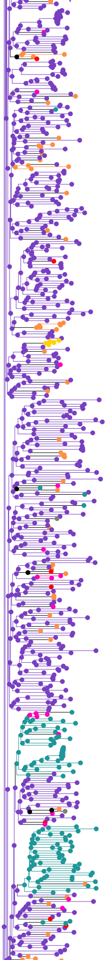

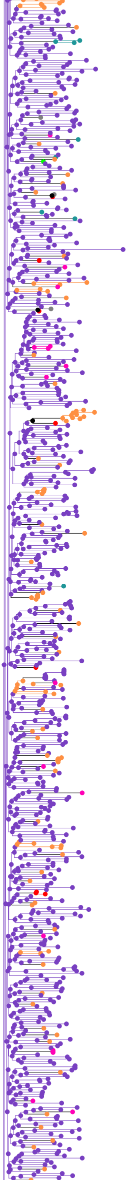

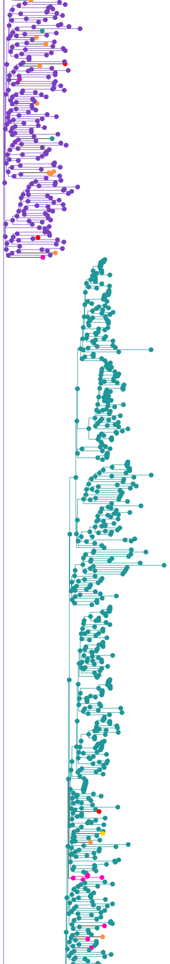

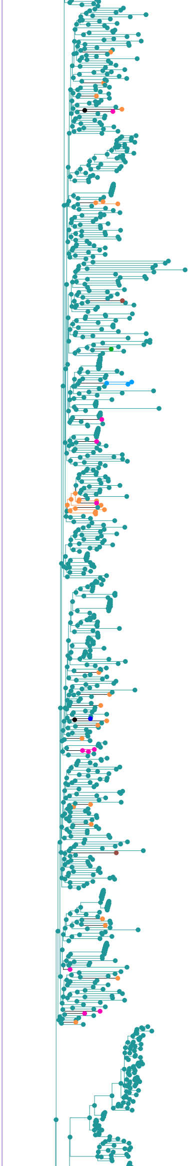

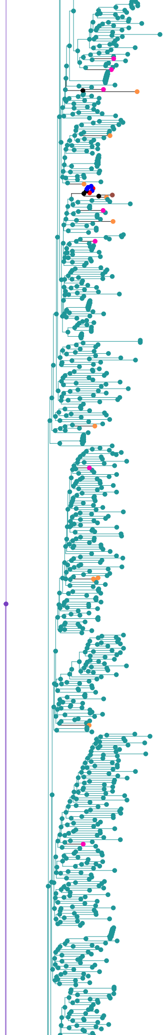

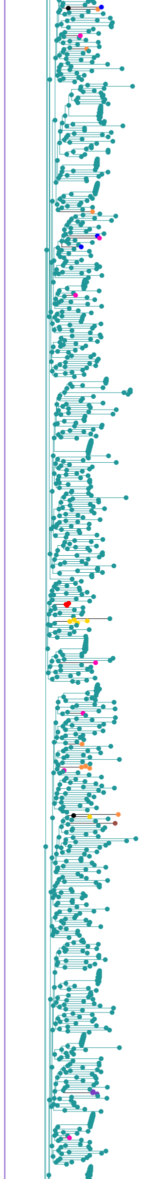

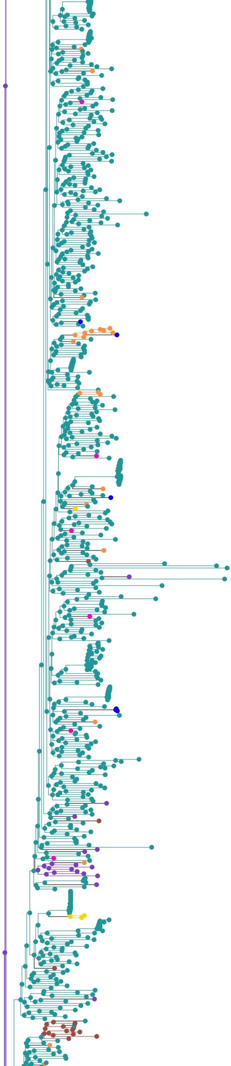

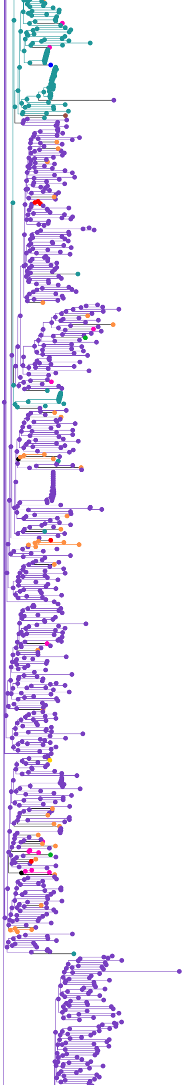

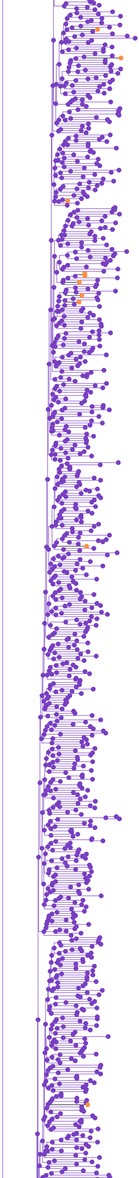

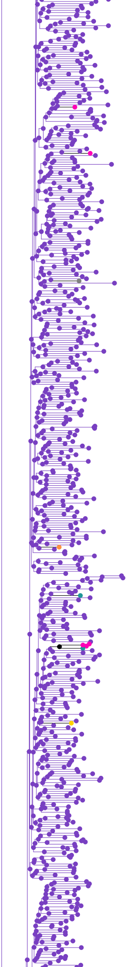

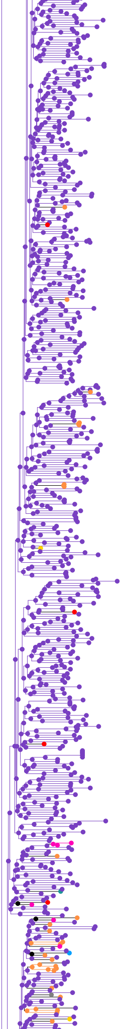

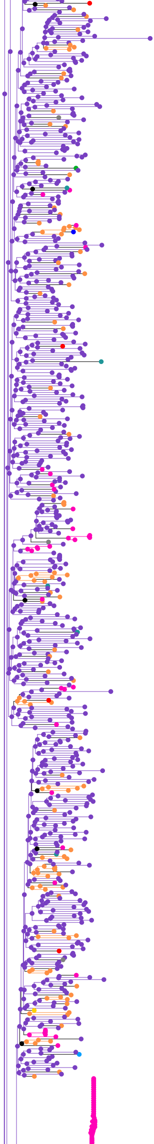

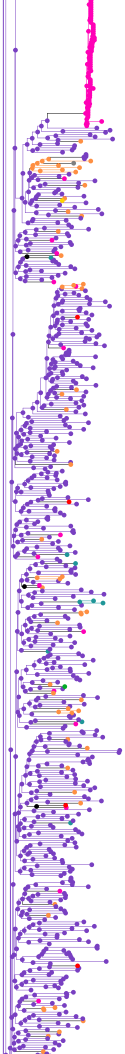

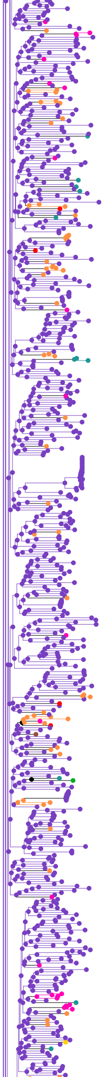

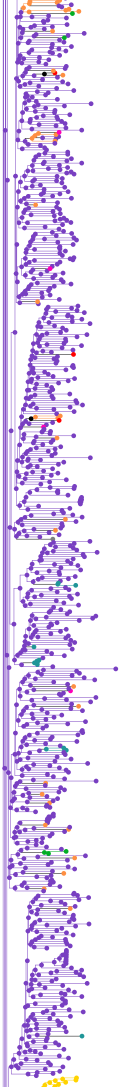

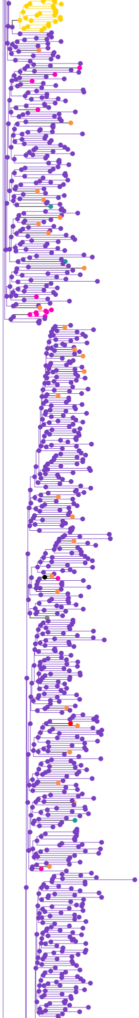

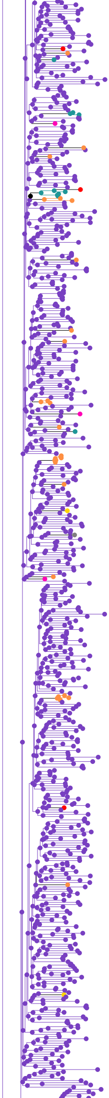

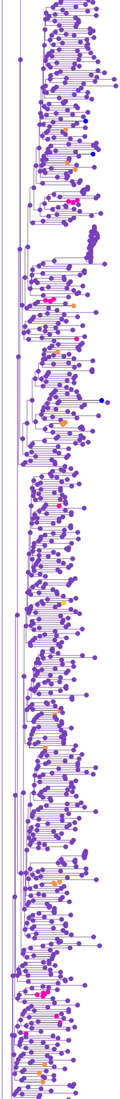

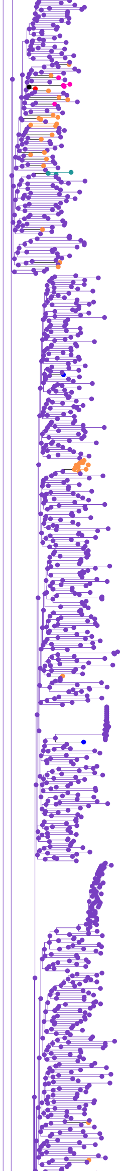

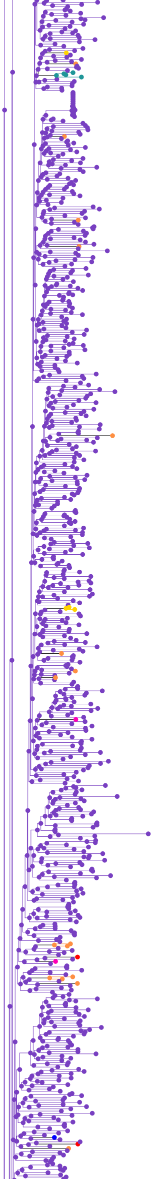

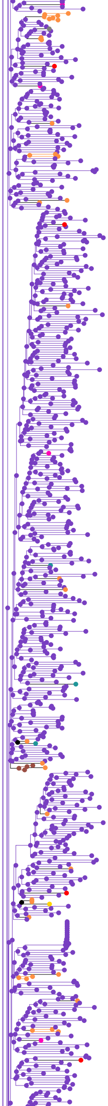

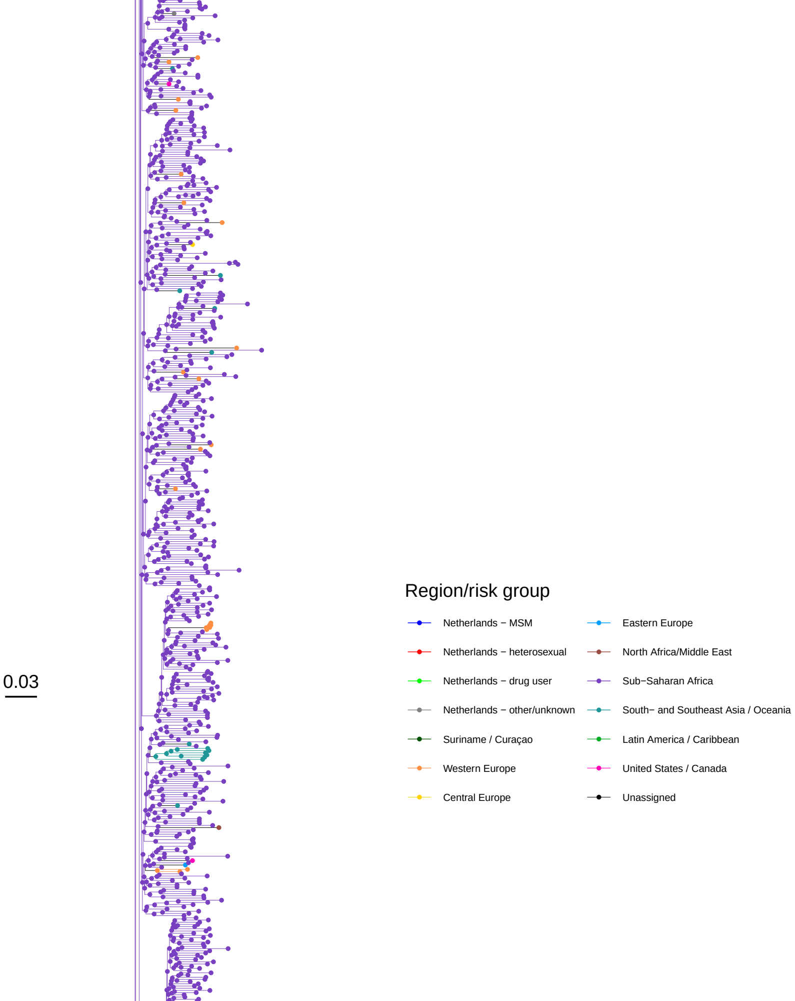
0.03
Region/risk group
Netherlands − MSM
Netherlands − heterosexual
Netherlands − drug user
Netherlands − other/unknown
Suriname / Curaçao
Western Europe
Central Europe
Eastern Europe
North Africa/Middle East
Sub−Saharan Africa
South− and Southeast Asia / Oceania
Latin America / Caribbean
United States / Canada
Unassigned

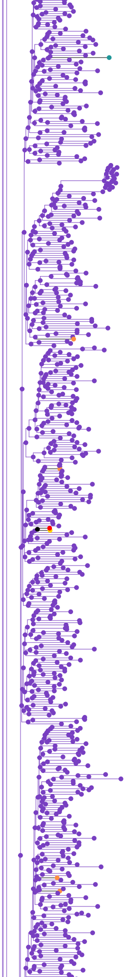

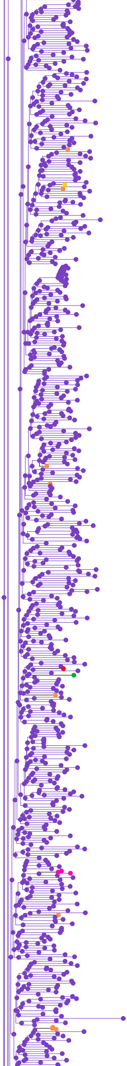

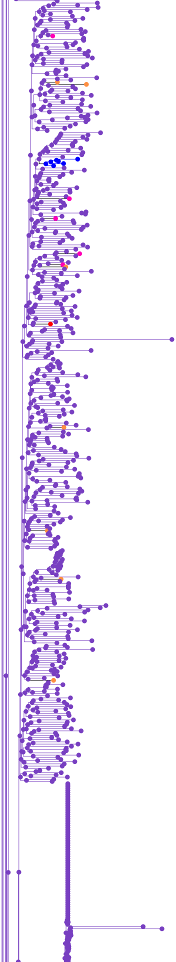

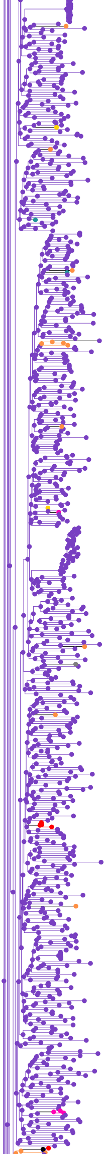

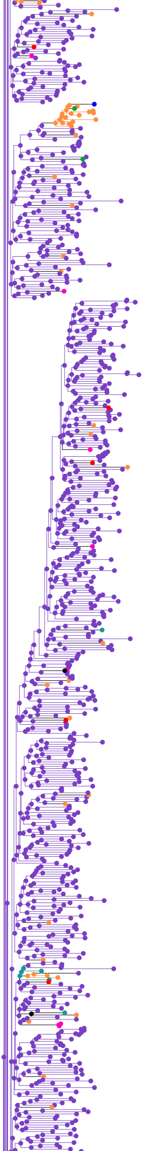

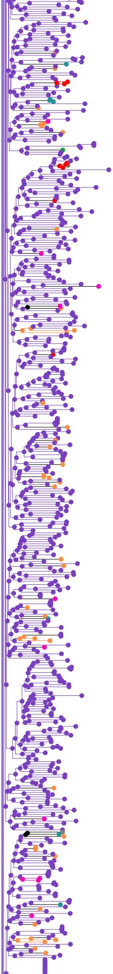

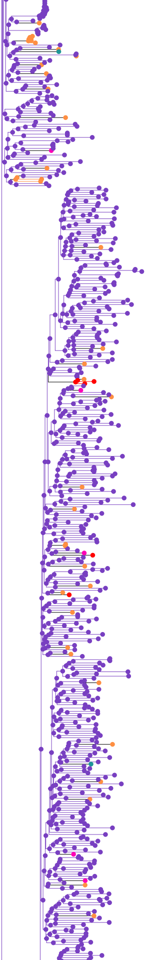

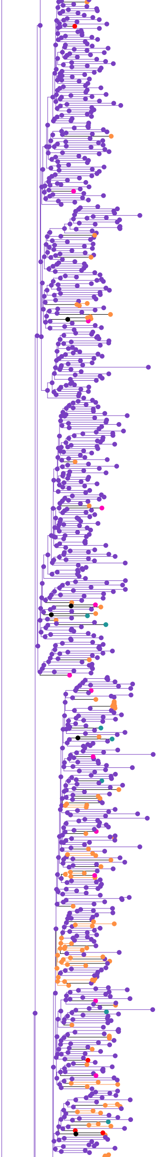

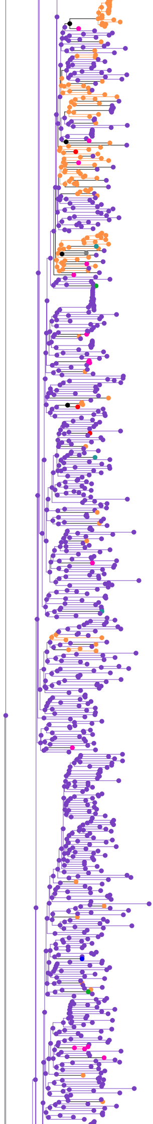

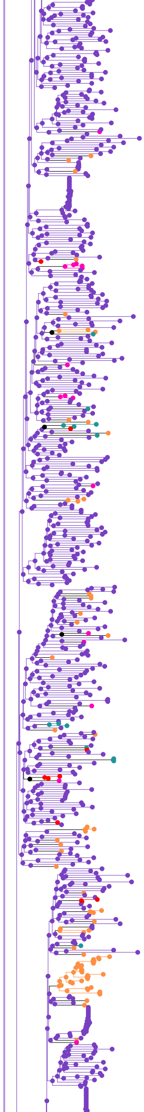

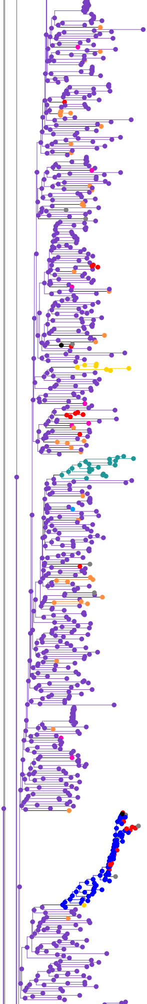

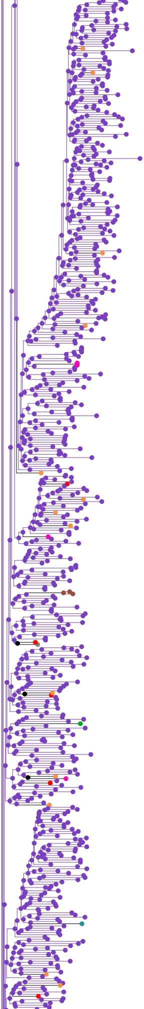

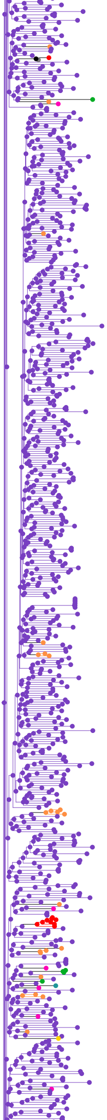

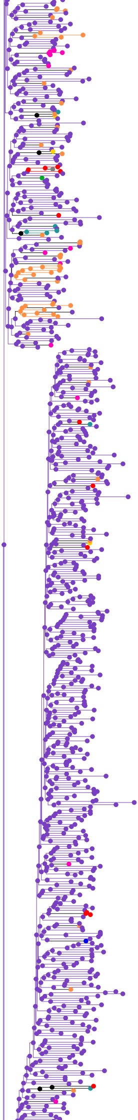

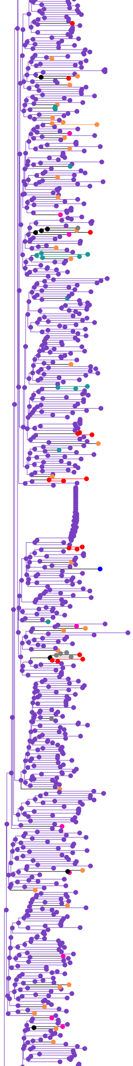

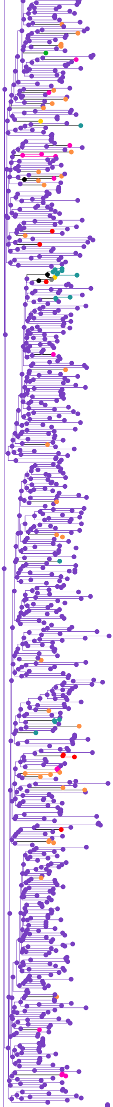

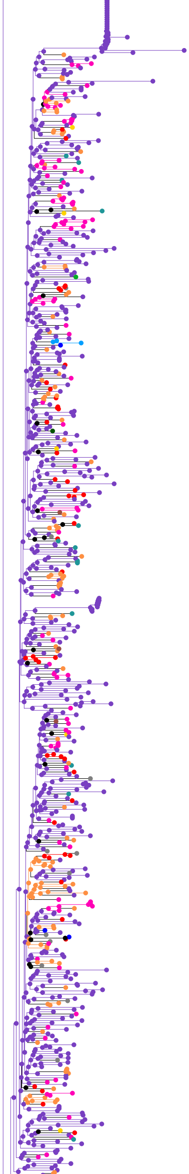

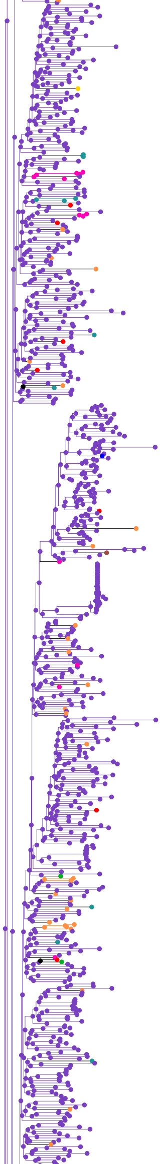

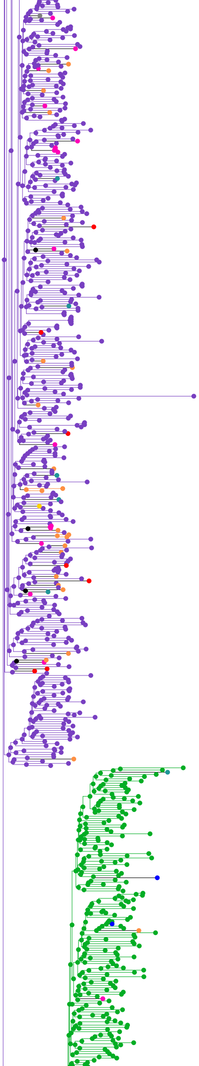

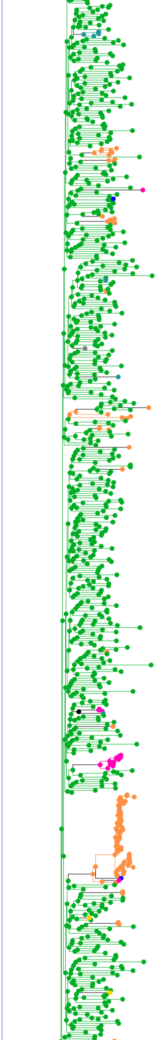

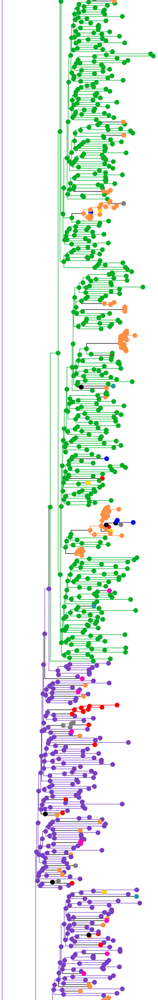

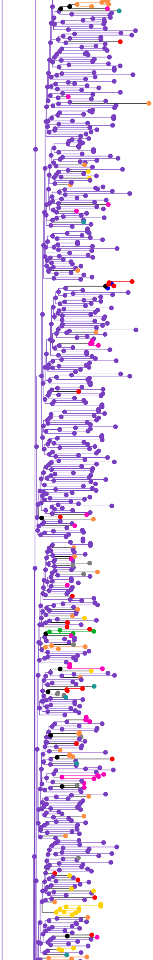

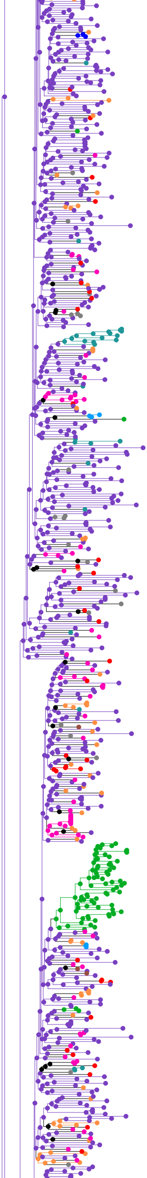

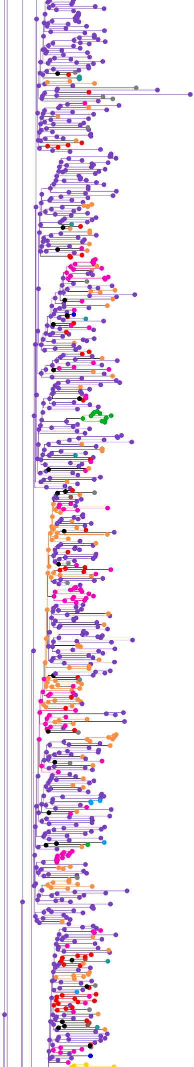

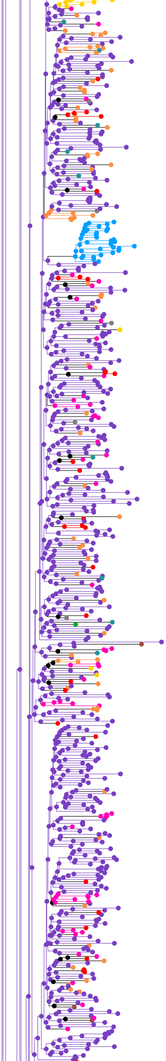

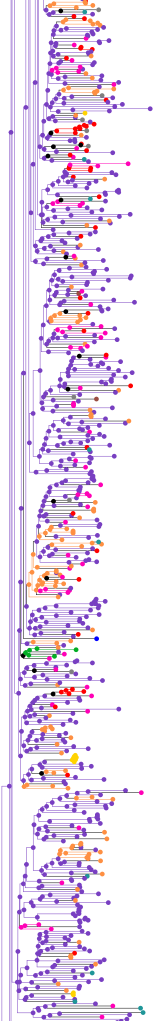

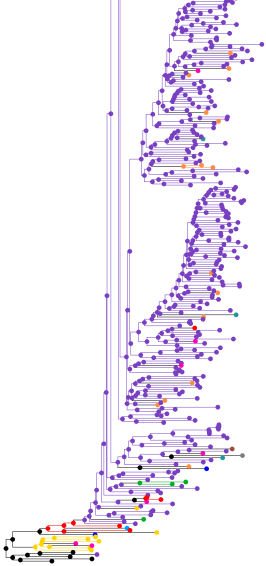

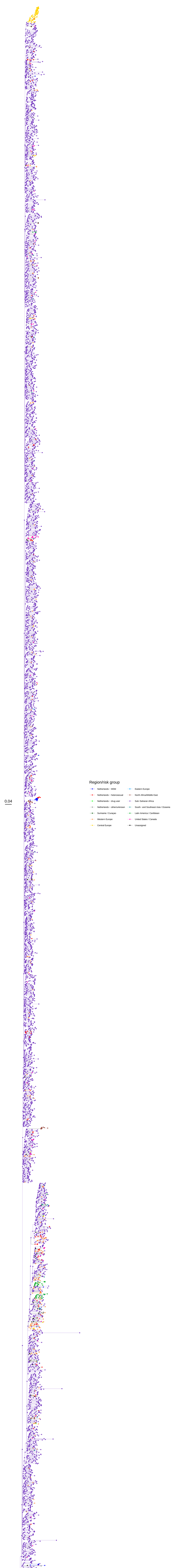
0.04
Region/risk group
Netherlands − MSM
Netherlands − heterosexual
Netherlands − drug user
Netherlands − other/unknown
Suriname / Curaçao
Western Europe
Central Europe
Eastern Europe
North Africa/Middle East
Sub−Saharan Africa
South− and Southeast Asia / Oceania
Latin America / Caribbean
United States / Canada
Unassigned

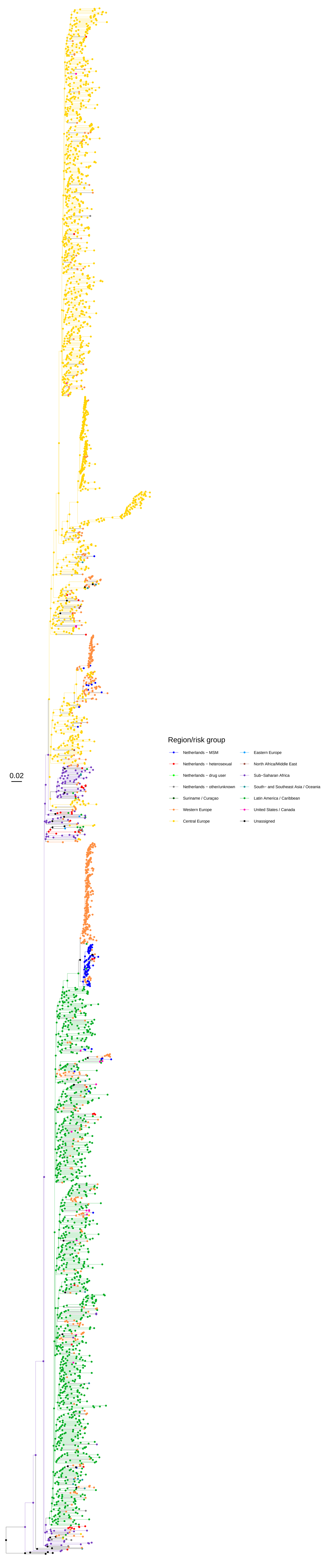
0.02
Region/risk group
Netherlands − MSM
Netherlands − heterosexual
Netherlands − drug user
Netherlands − other/unknown
Suriname / Curaçao
Western Europe
Central Europe
Eastern Europe
North Africa/Middle East
Sub−Saharan Africa
South− and Southeast Asia / Oceania
Latin America / Caribbean
United States / Canada
Unassigned

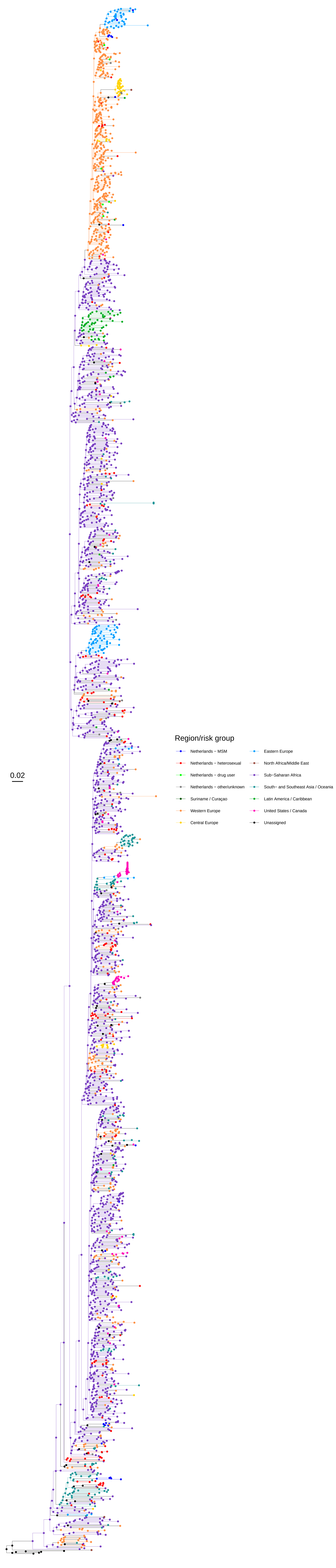
0.02
Region/risk group
Netherlands − MSM
Netherlands − heterosexual
Netherlands − drug user
Netherlands − other/unknown
Suriname / Curaçao
Western Europe
Central Europe
Eastern Europe
North Africa/Middle East
Sub−Saharan Africa
South− and Southeast Asia / Oceania
Latin America / Caribbean
United States / Canada
Unassigned

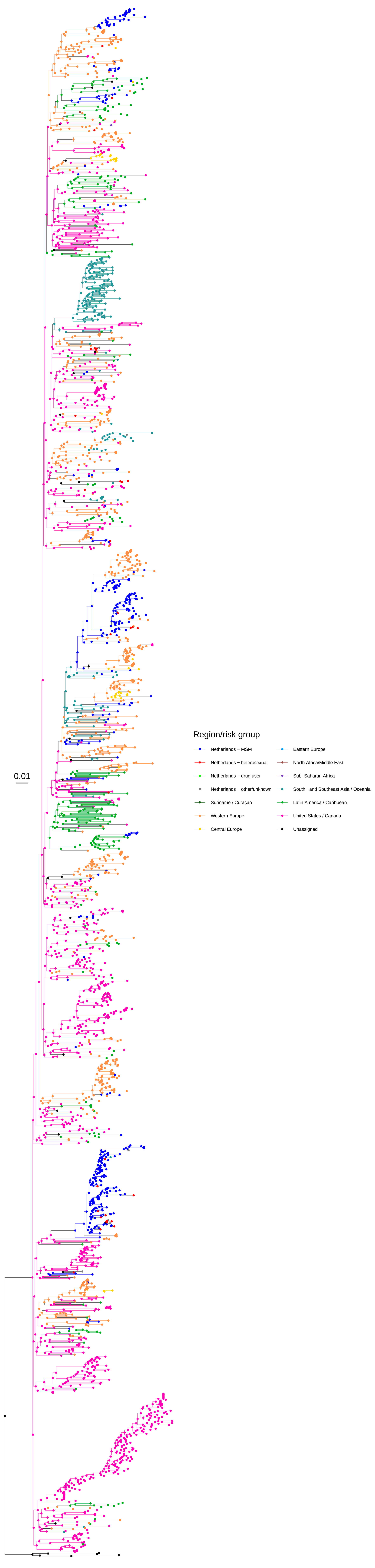
0.01
Region/risk group
Netherlands − MSM
Netherlands − heterosexual
Netherlands − drug user
Netherlands − other/unknown
Suriname / Curaçao
Western Europe
Central Europe
Eastern Europe
North Africa/Middle East
Sub−Saharan Africa
South− and Southeast Asia / Oceania
Latin America / Caribbean
United States / Canada
Unassigned

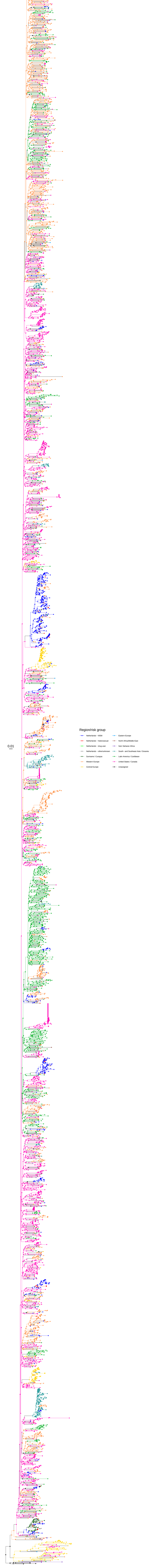
0.01
Region/risk group
Netherlands − MSM
Netherlands − heterosexual
Netherlands − drug user
Netherlands − other/unknown
Suriname / Curaçao
Western Europe
Central Europe
Eastern Europe
North Africa/Middle East
Sub−Saharan Africa
South− and Southeast Asia / Oceania
Latin America / Caribbean
United States / Canada
Unassigned

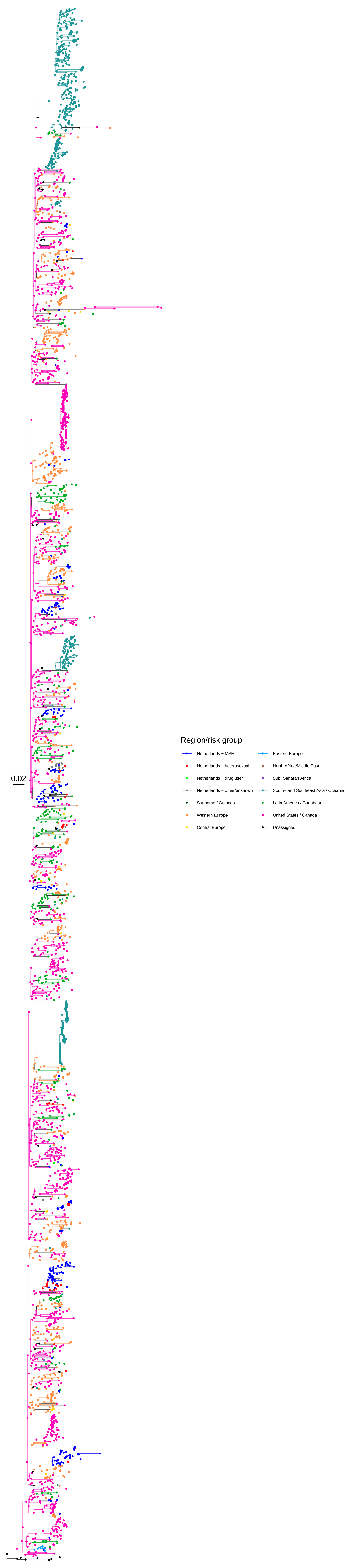
0.02
Region/risk group
Netherlands − MSM
Netherlands − heterosexual
Netherlands − drug user
Netherlands − other/unknown
Suriname / Curaçao
Western Europe
Central Europe
Eastern Europe
North Africa/Middle East
Sub−Saharan Africa
South− and Southeast Asia / Oceania
Latin America / Caribbean
United States / Canada
Unassigned

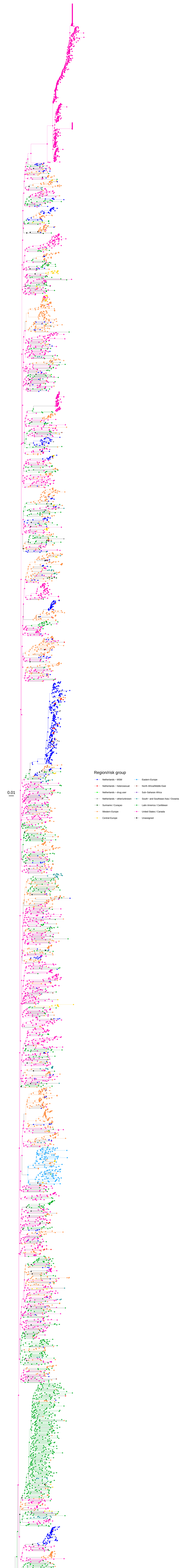
0.01
Region/risk group
Netherlands − MSM
Netherlands − heterosexual
Netherlands − drug user
Netherlands − other/unknown
Suriname / Curaçao
Western Europe
Central Europe
Eastern Europe
North Africa/Middle East
Sub−Saharan Africa
South− and Southeast Asia / Oceania
Latin America / Caribbean
United States / Canada
Unassigned

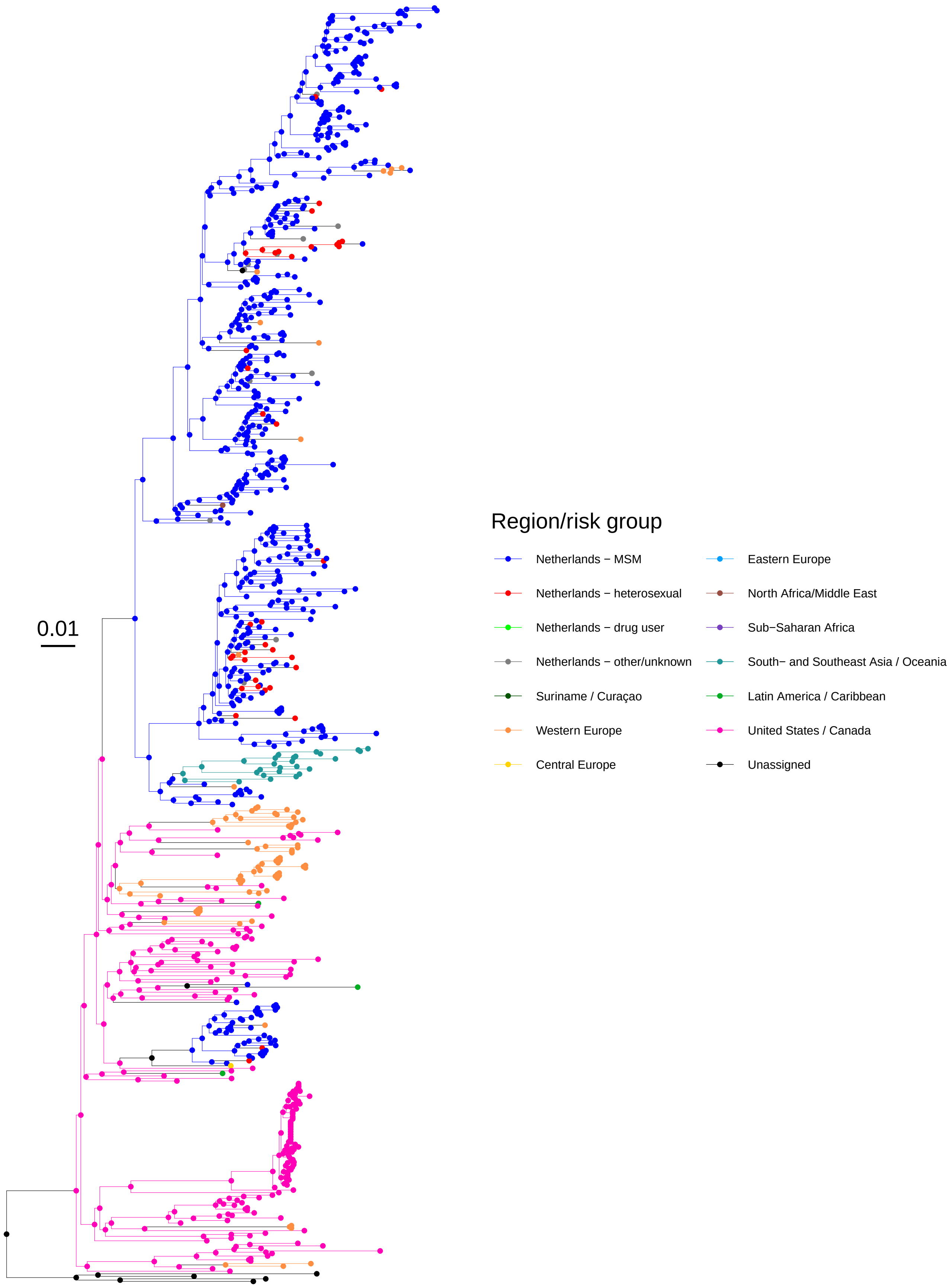
0.01
Region/risk group
Netherlands − MSM
Netherlands − heterosexual
Netherlands − drug user
Netherlands − other/unknown
Suriname / Curaçao
Western Europe
Central Europe
Eastern Europe
North Africa/Middle East
Sub−Saharan Africa
South− and Southeast Asia / Oceania
Latin America / Caribbean
United States / Canada
Unassigned

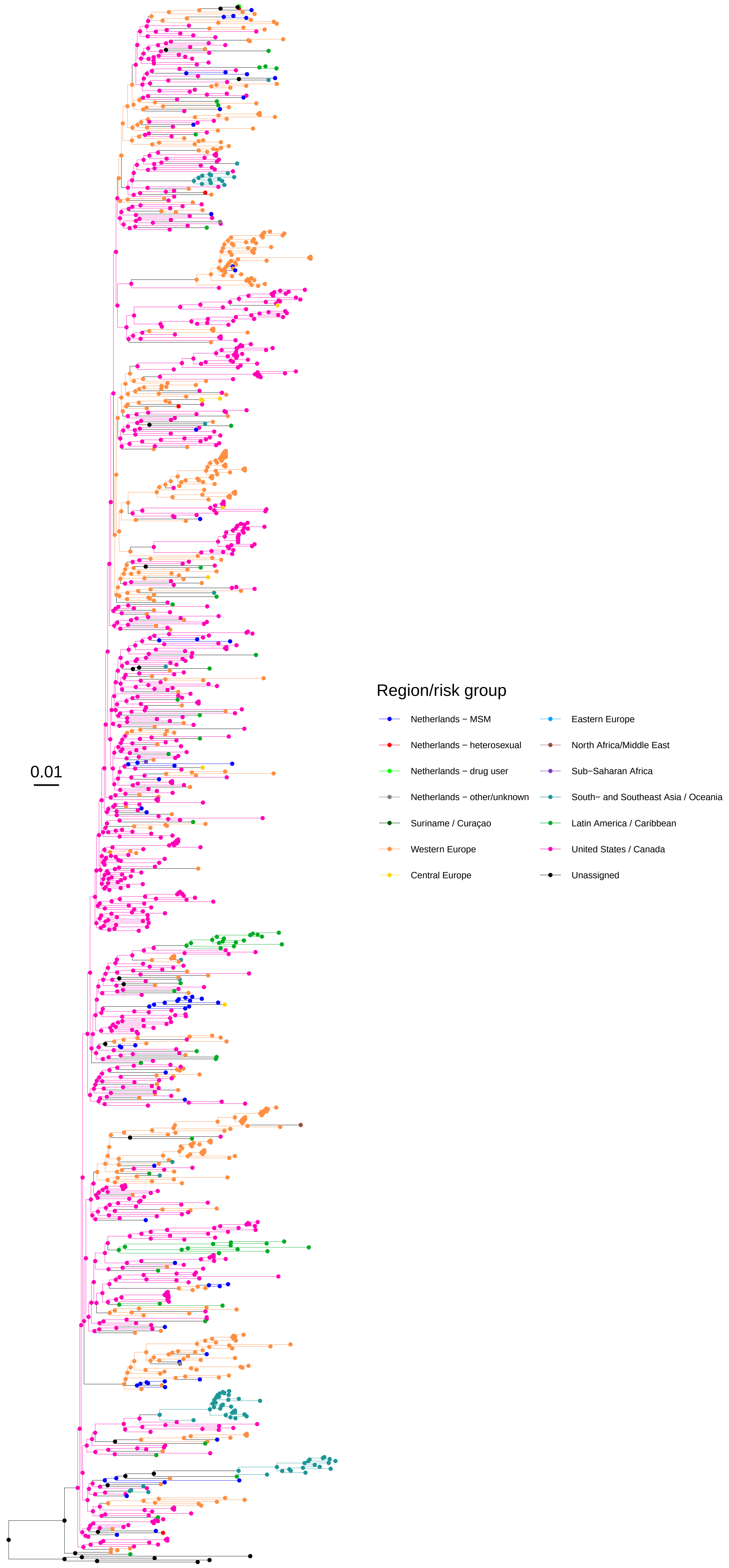
0.01
Region/risk group
Netherlands − MSM
Netherlands − heterosexual
Netherlands − drug user
Netherlands − other/unknown
Suriname / Curaçao
Western Europe
Central Europe
Eastern Europe
North Africa/Middle East
Sub−Saharan Africa
South− and Southeast Asia / Oceania
Latin America / Caribbean
United States / Canada
Unassigned

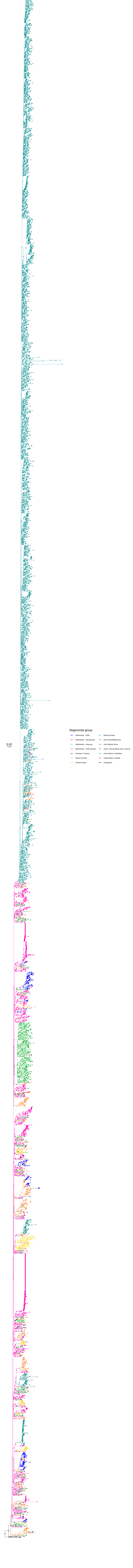
0.02
Region/risk group
Netherlands − MSM
Netherlands − heterosexual
Netherlands − drug user
Netherlands − other/unknown
Suriname / Curaçao
Western Europe
Central Europe
Eastern Europe
North Africa/Middle East
Sub−Saharan Africa
South− and Southeast Asia / Oceania
Latin America / Caribbean
United States / Canada
Unassigned

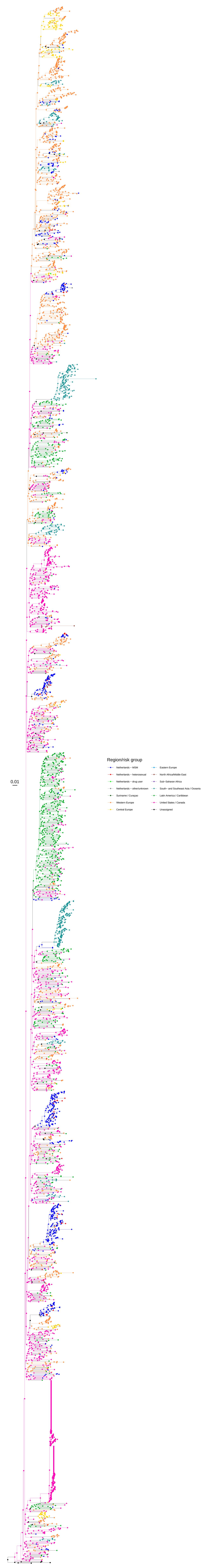
0.01
Region/risk group
Netherlands − MSM
Netherlands − heterosexual
Netherlands − drug user
Netherlands − other/unknown
Suriname / Curaçao
Western Europe
Central Europe
Eastern Europe
North Africa/Middle East
Sub−Saharan Africa
South− and Southeast Asia / Oceania
Latin America / Caribbean
United States / Canada
Unassigned

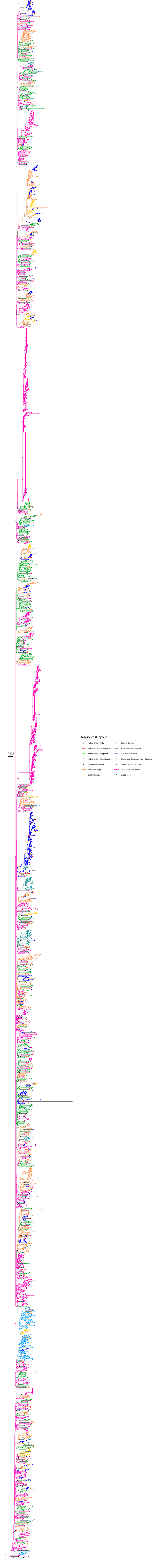
0.02
Region/risk group
Netherlands − MSM
Netherlands − heterosexual
Netherlands − drug user
Netherlands − other/unknown
Suriname / Curaçao
Western Europe
Central Europe
Eastern Europe
North Africa/Middle East
Sub−Saharan Africa
South− and Southeast Asia / Oceania
Latin America / Caribbean
United States / Canada
Unassigned

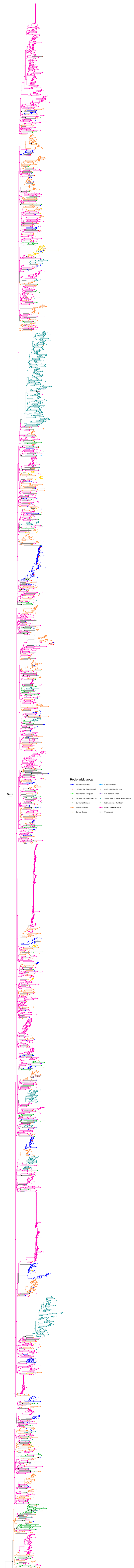

0.01
Region/risk group
Netherlands − MSM
Netherlands − heterosexual
Netherlands − drug user
Netherlands − other/unknown
Suriname / Curaçao
Western Europe
Central Europe
Eastern Europe
North Africa/Middle East
Sub−Saharan Africa
South− and Southeast Asia / Oceania
Latin America / Caribbean
United States / Canada
Unassigned

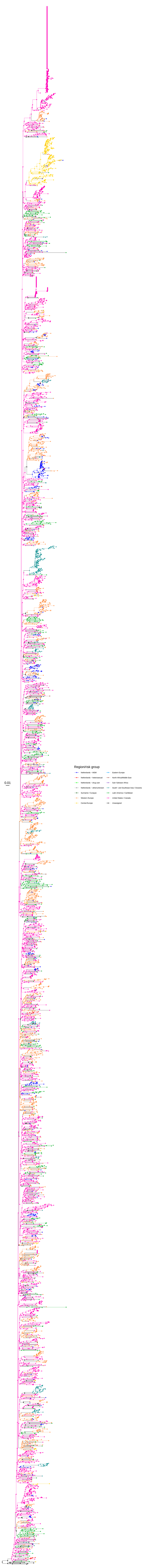
0.01
Region/risk group
Netherlands − MSM
Netherlands − heterosexual
Netherlands − drug user
Netherlands − other/unknown
Suriname / Curaçao
Western Europe
Central Europe
Eastern Europe
North Africa/Middle East
Sub−Saharan Africa
South− and Southeast Asia / Oceania
Latin America / Caribbean
United States / Canada
Unassigned

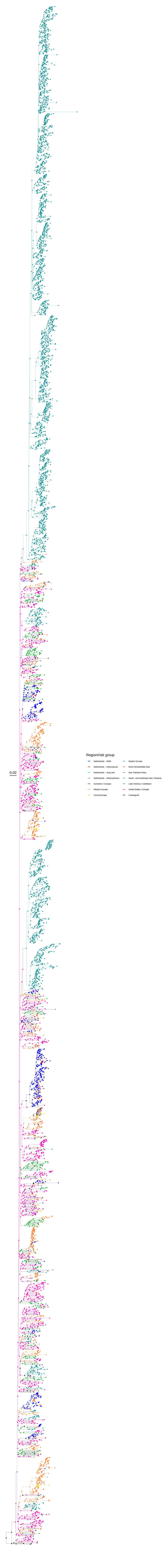
0.02
Region/risk group
Netherlands − MSM
Netherlands − heterosexual
Netherlands − drug user
Netherlands − other/unknown
Suriname / Curaçao
Western Europe
Central Europe
Eastern Europe
North Africa/Middle East
Sub−Saharan Africa
South− and Southeast Asia / Oceania
Latin America / Caribbean
United States / Canada
Unassigned

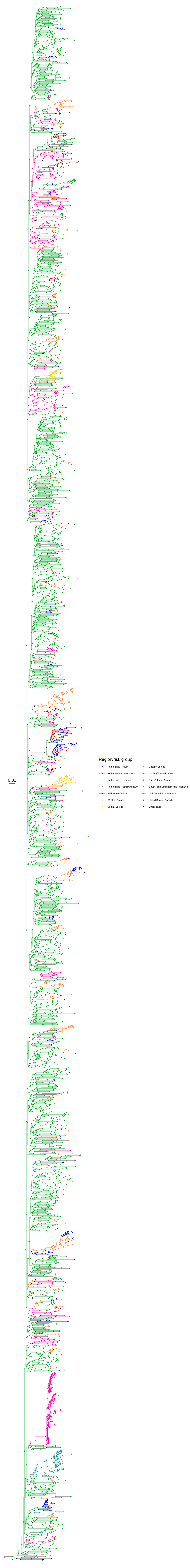
0.01
Region/risk group
Netherlands − MSM
Netherlands − heterosexual
Netherlands − drug user
Netherlands − other/unknown
Suriname / Curaçao
Western Europe
Central Europe
Eastern Europe
North Africa/Middle East
Sub−Saharan Africa
South− and Southeast Asia / Oceania
Latin America / Caribbean
United States / Canada
Unassigned

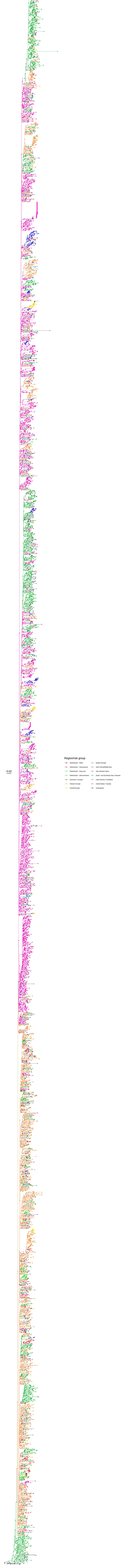
0.02
Region/risk group
Netherlands − MSM
Netherlands − heterosexual
Netherlands − drug user
Netherlands − other/unknown
Suriname / Curaçao
Western Europe
Central Europe
Eastern Europe
North Africa/Middle East
Sub−Saharan Africa
South− and Southeast Asia / Oceania
Latin America / Caribbean
United States / Canada
Unassigned

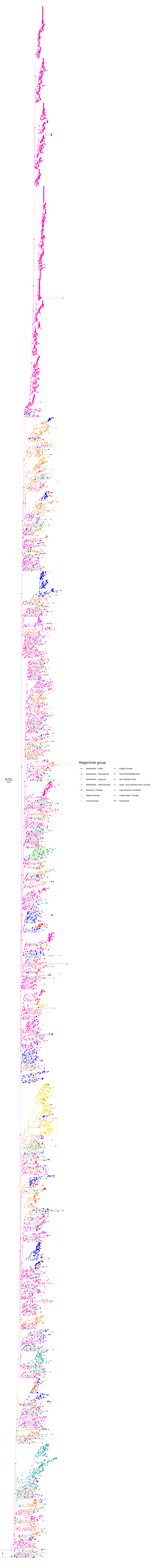

0.01
Region/risk group
Netherlands − MSM
Netherlands − heterosexual
Netherlands − drug user
Netherlands − other/unknown
Suriname / Curaçao
Western Europe
Central Europe
Eastern Europe
North Africa/Middle East
Sub−Saharan Africa
South− and Southeast Asia / Oceania
Latin America / Caribbean
United States / Canada
Unassigned

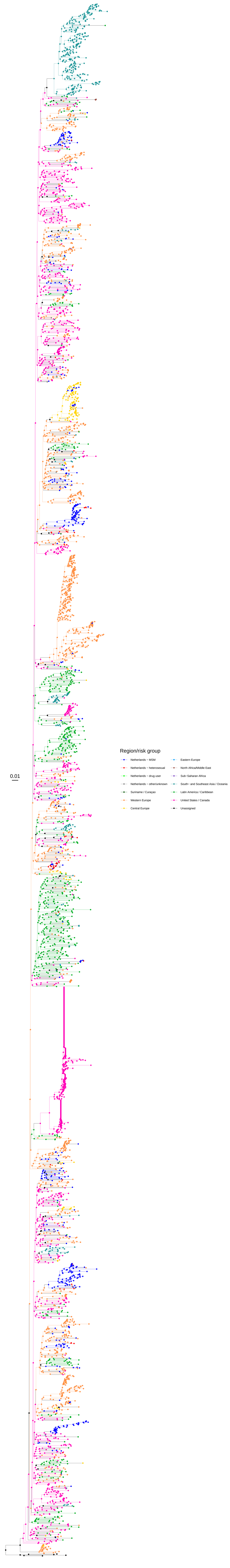
0.01
Region/risk group
Netherlands − MSM
Netherlands − heterosexual
Netherlands − drug user
Netherlands − other/unknown
Suriname / Curaçao
Western Europe
Central Europe
Eastern Europe
North Africa/Middle East
Sub−Saharan Africa
South− and Southeast Asia / Oceania
Latin America / Caribbean
United States / Canada
Unassigned

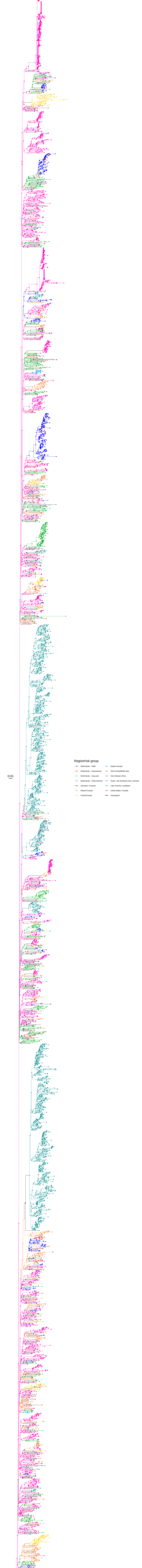
0.01
Region/risk group
Netherlands − MSM
Netherlands − heterosexual
Netherlands − drug user
Netherlands − other/unknown
Suriname / Curaçao
Western Europe
Central Europe
Eastern Europe
North Africa/Middle East
Sub−Saharan Africa
South− and Southeast Asia / Oceania
Latin America / Caribbean
United States / Canada
Unassigned

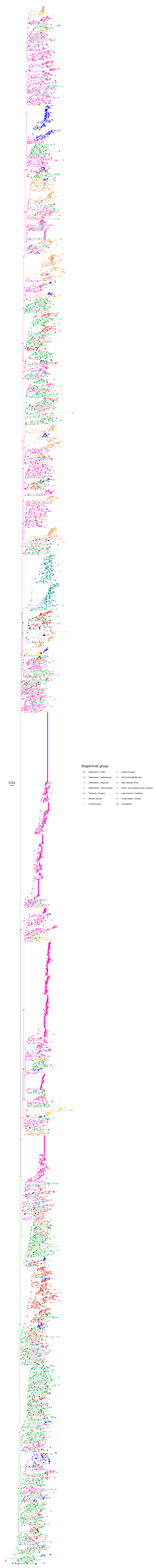
0.01
Region/risk group
Netherlands − MSM
Netherlands − heterosexual
Netherlands − drug user
Netherlands − other/unknown
Suriname / Curaçao
Western Europe
Central Europe
Eastern Europe
North Africa/Middle East
Sub−Saharan Africa
South− and Southeast Asia / Oceania
Latin America / Caribbean
United States / Canada
Unassigned

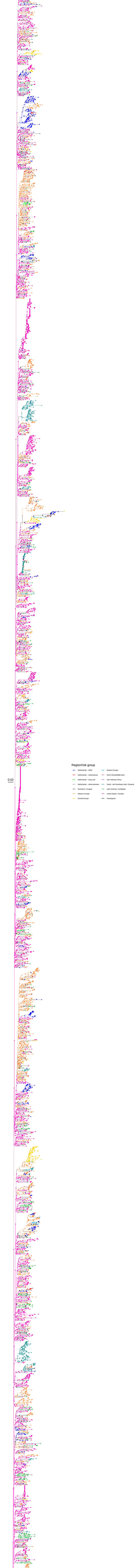

0.02
Region/risk group
Netherlands − MSM
Netherlands − heterosexual
Netherlands − drug user
Netherlands − other/unknown
Suriname / Curaçao
Western Europe
Central Europe
Eastern Europe
North Africa/Middle East
Sub−Saharan Africa
South− and Southeast Asia / Oceania
Latin America / Caribbean
United States / Canada
Unassigned

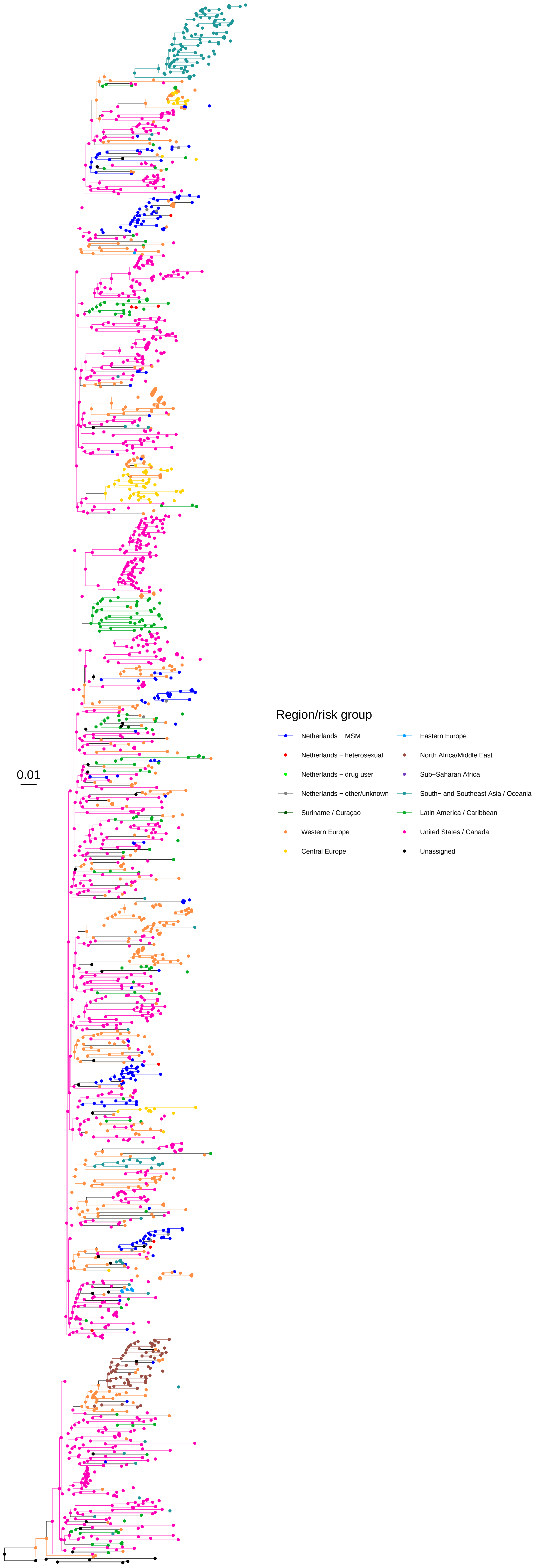
0.01
Region/risk group
Netherlands − MSM
Netherlands − heterosexual
Netherlands − drug user
Netherlands − other/unknown
Suriname / Curaçao
Western Europe
Central Europe
Eastern Europe
North Africa/Middle East
Sub−Saharan Africa
South− and Southeast Asia / Oceania
Latin America / Caribbean
United States / Canada
Unassigned

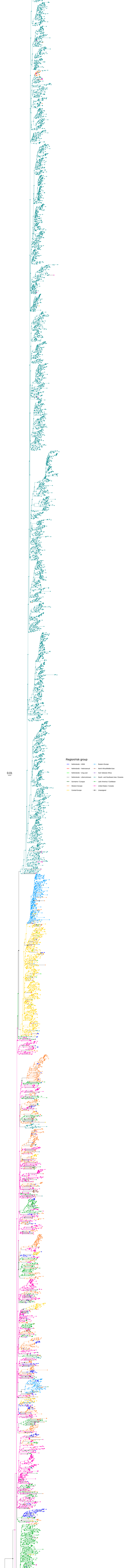
0.01
Region/risk group
Netherlands − MSM
Netherlands − heterosexual
Netherlands − drug user
Netherlands − other/unknown
Suriname / Curaçao
Western Europe
Central Europe
Eastern Europe
North Africa/Middle East
Sub−Saharan Africa
South− and Southeast Asia / Oceania
Latin America / Caribbean
United States / Canada
Unassigned

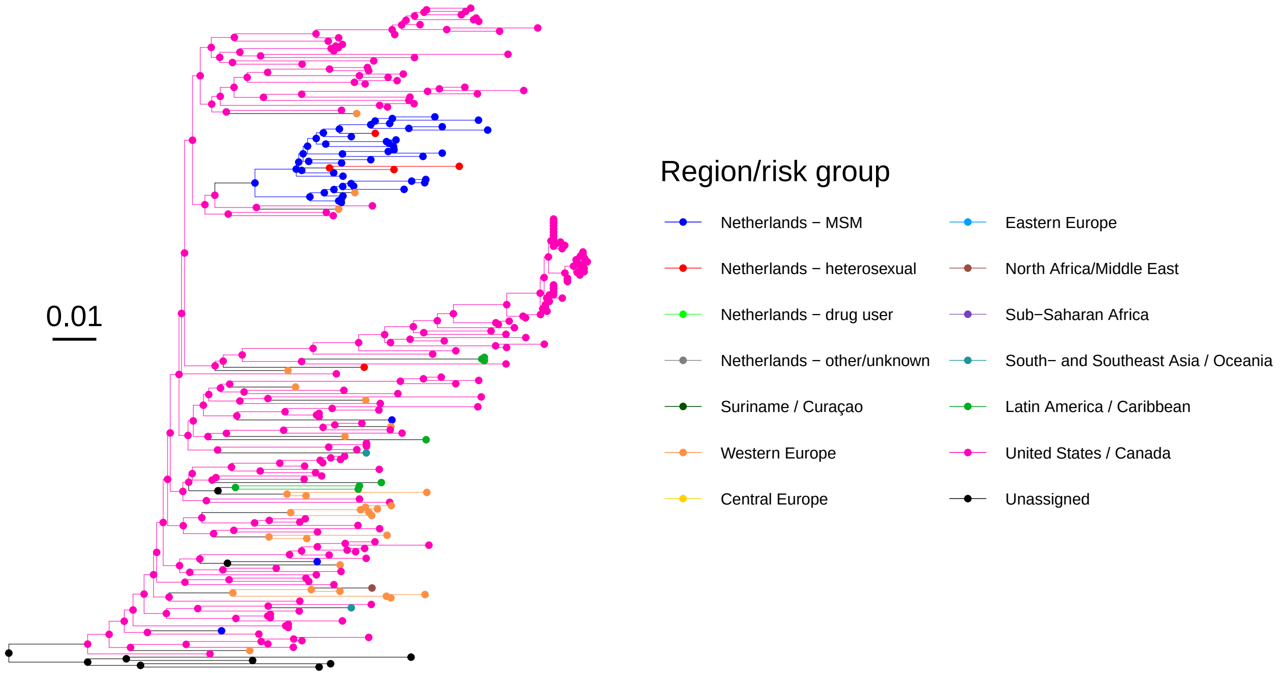
0.01
Region/risk group
Netherlands − MSM
Netherlands − heterosexual
Netherlands − drug user
Netherlands − other/unknown
Suriname / Curaçao
Western Europe
Central Europe
Eastern Europe
North Africa/Middle East
Sub−Saharan Africa
South− and Southeast Asia / Oceania
Latin America / Caribbean
United States / Canada
Unassigned

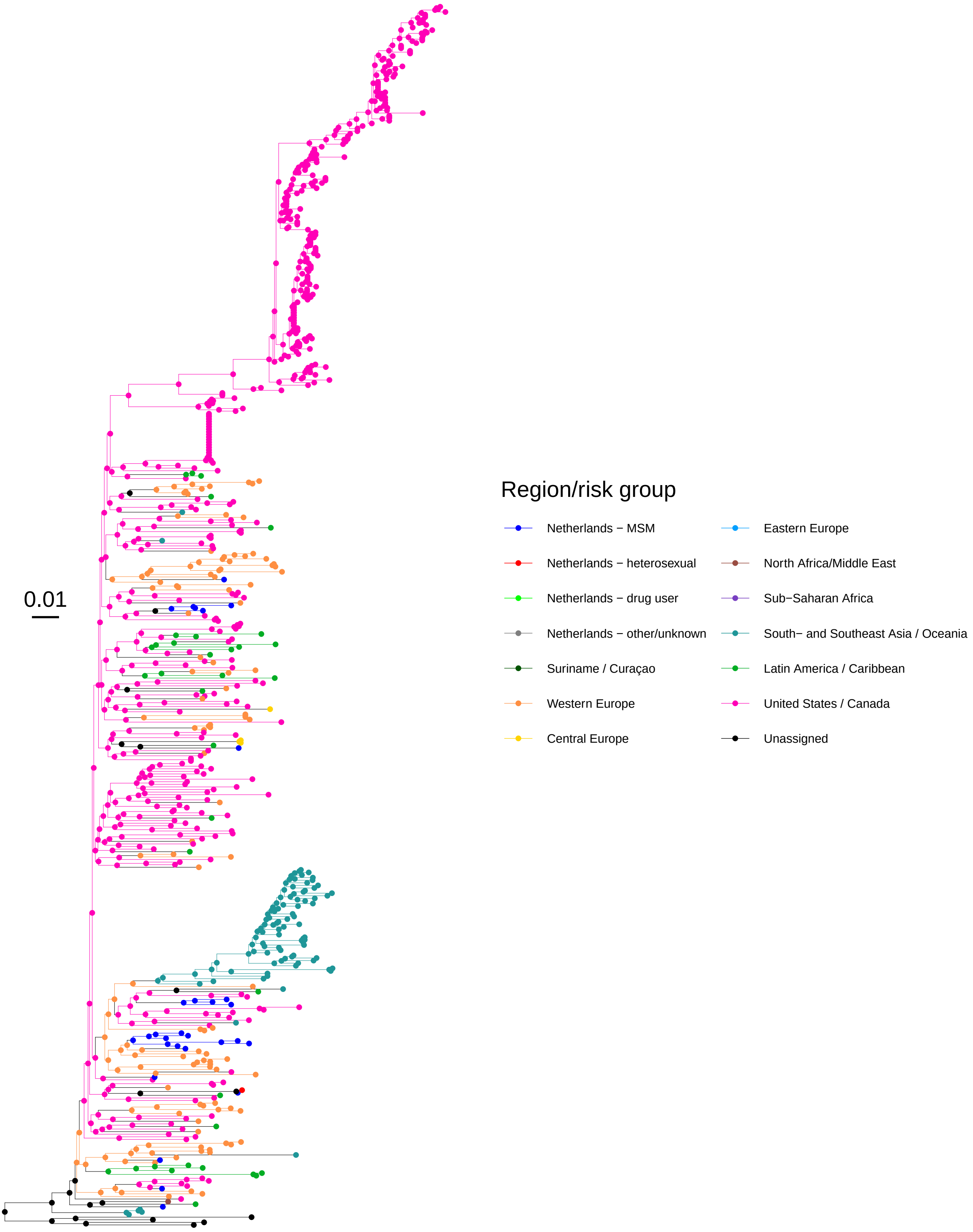
0.01
Region/risk group
Netherlands − MSM
Netherlands − heterosexual
Netherlands − drug user
Netherlands − other/unknown
Suriname / Curaçao
Western Europe
Central Europe
Eastern Europe
North Africa/Middle East
Sub−Saharan Africa
South− and Southeast Asia / Oceania
Latin America / Caribbean
United States / Canada
Unassigned

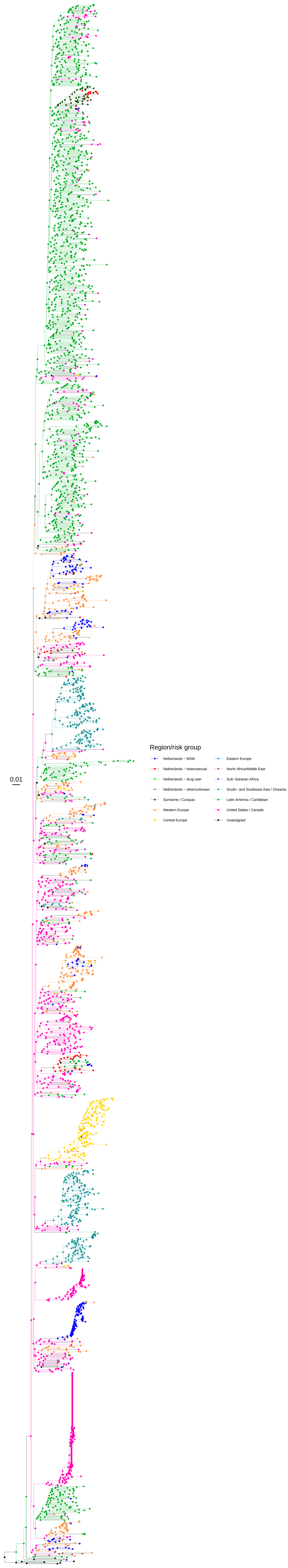
0.01
Region/risk group
Netherlands − MSM
Netherlands − heterosexual
Netherlands − drug user
Netherlands − other/unknown
Suriname / Curaçao
Western Europe
Central Europe
Eastern Europe
North Africa/Middle East
Sub−Saharan Africa
South− and Southeast Asia / Oceania
Latin America / Caribbean
United States / Canada
Unassigned

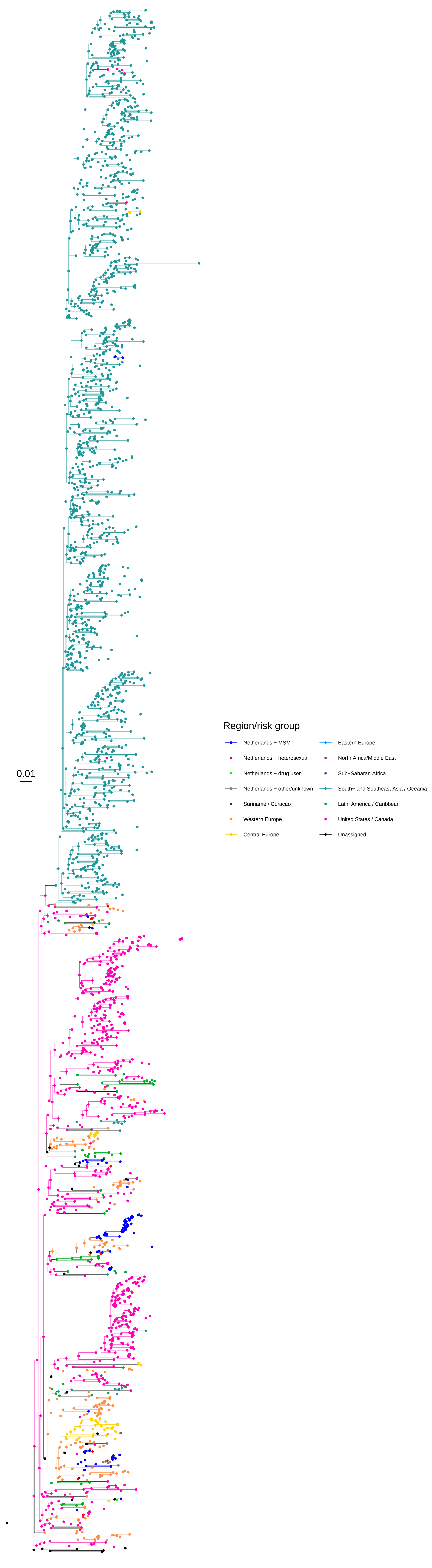
0.01
Region/risk group
Netherlands − MSM
Netherlands − heterosexual
Netherlands − drug user
Netherlands − other/unknown
Suriname / Curaçao
Western Europe
Central Europe
Eastern Europe
North Africa/Middle East
Sub−Saharan Africa
South− and Southeast Asia / Oceania
Latin America / Caribbean
United States / Canada
Unassigned

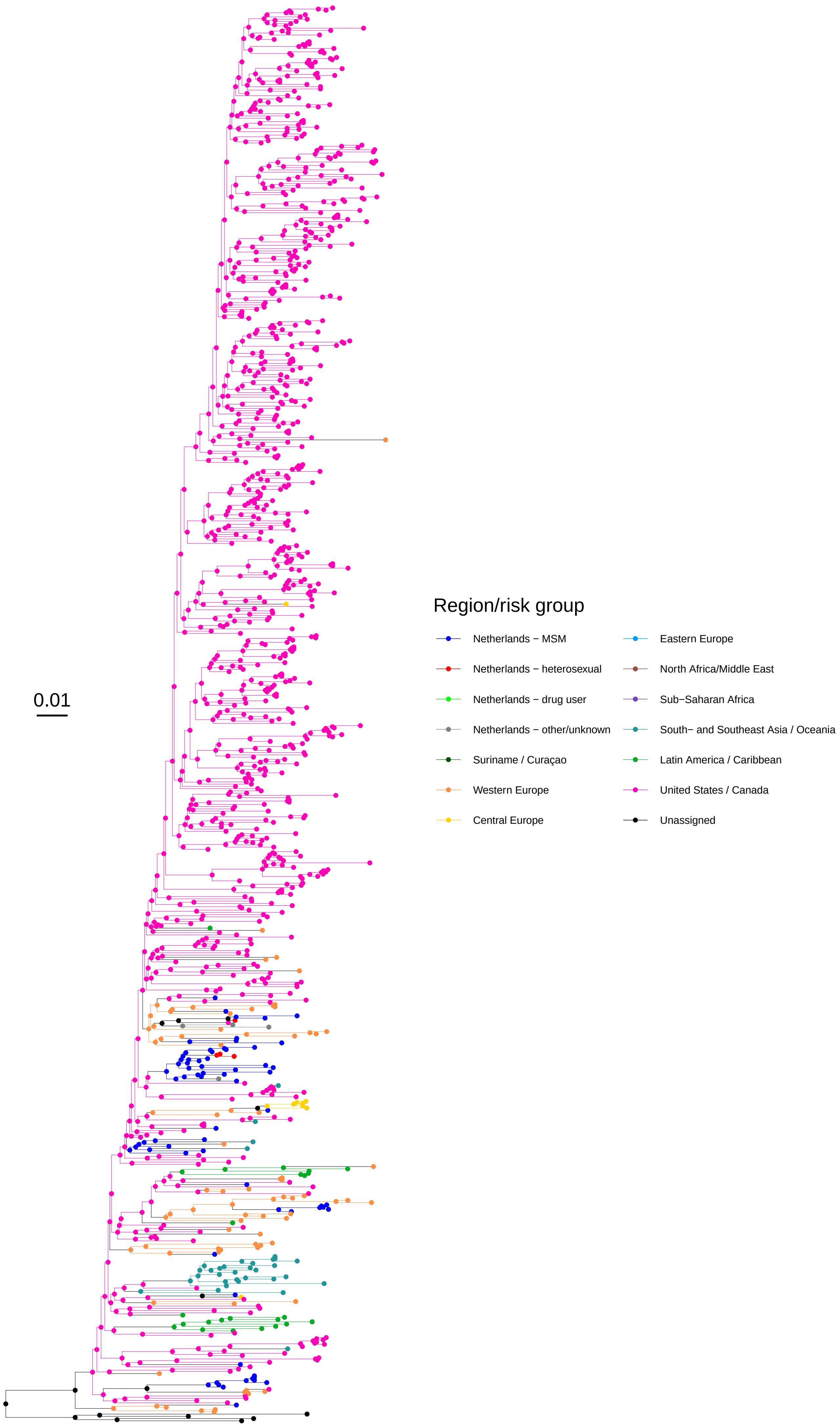
0.01
Region/risk group
Netherlands − MSM
Netherlands − heterosexual
Netherlands − drug user
Netherlands − other/unknown
Suriname / Curaçao
Western Europe
Central Europe
Eastern Europe
North Africa/Middle East
Sub−Saharan Africa
South− and Southeast Asia / Oceania
Latin America / Caribbean
United States / Canada
Unassigned

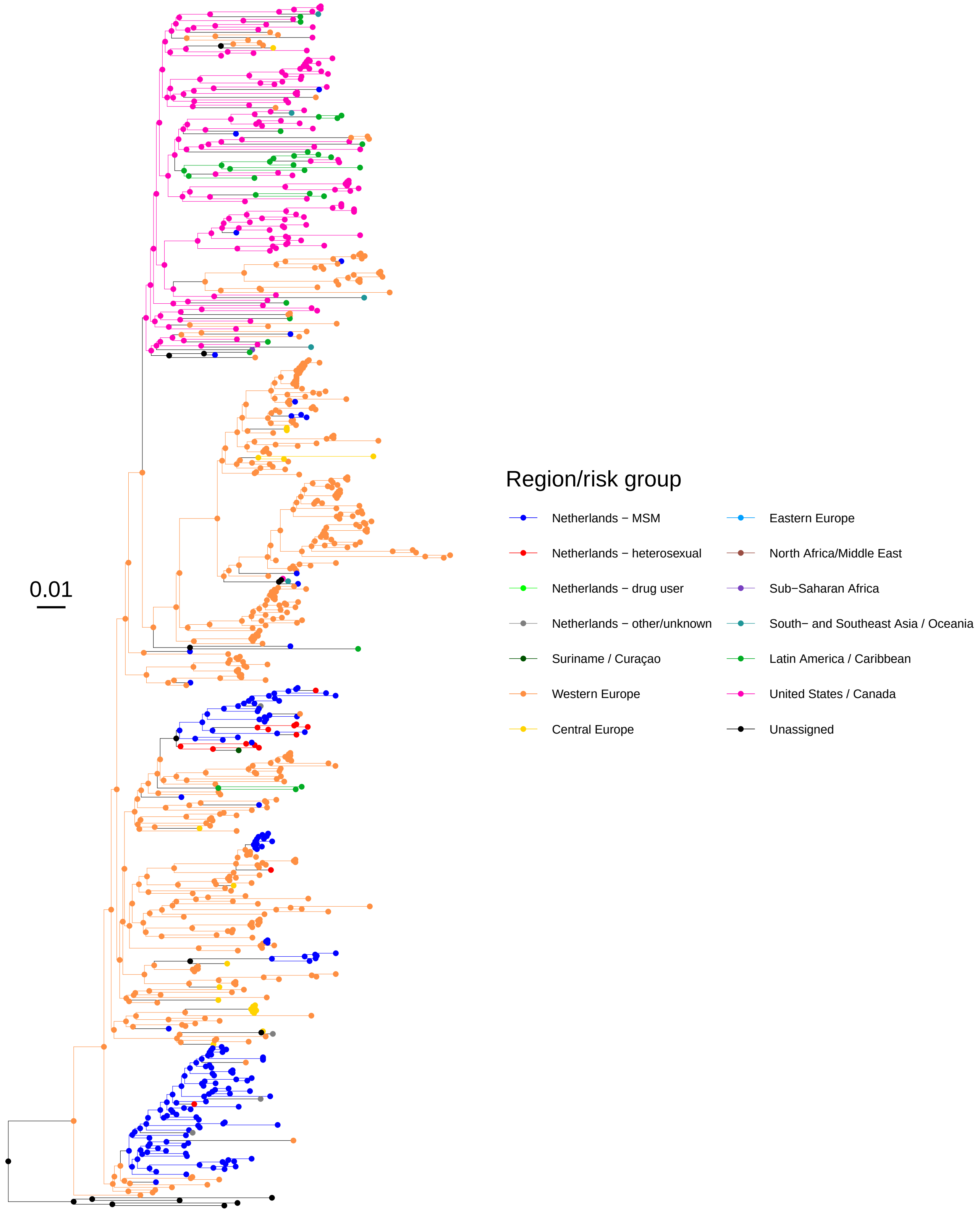

0.01
Region/risk group
Netherlands − MSM
Netherlands − heterosexual
Netherlands − drug user
Netherlands − other/unknown
Suriname / Curaçao
Western Europe
Central Europe
Eastern Europe
North Africa/Middle East
Sub−Saharan Africa
South− and Southeast Asia / Oceania
Latin America / Caribbean
United States / Canada
Unassigned

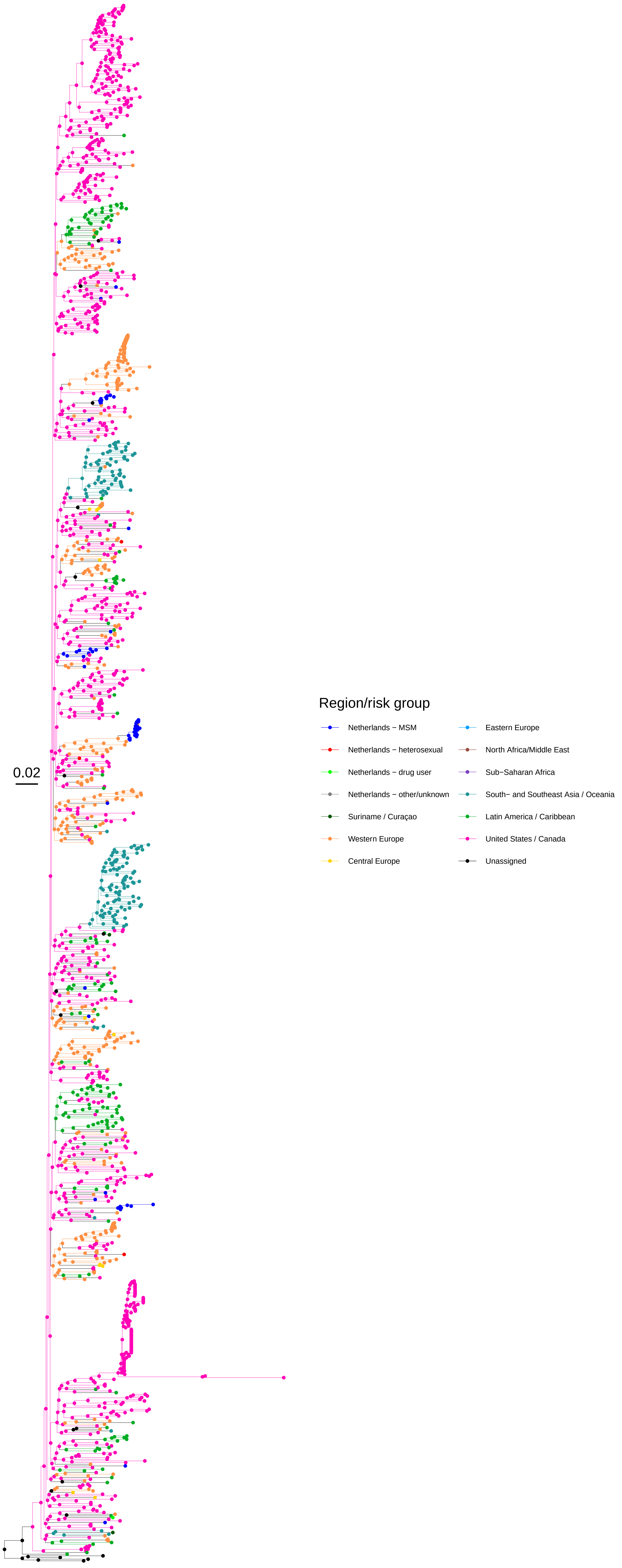
0.02
Region/risk group
Netherlands − MSM
Netherlands − heterosexual
Netherlands − drug user
Netherlands − other/unknown
Suriname / Curaçao
Western Europe
Central Europe
Eastern Europe
North Africa/Middle East
Sub−Saharan Africa
South− and Southeast Asia / Oceania
Latin America / Caribbean
United States / Canada
Unassigned

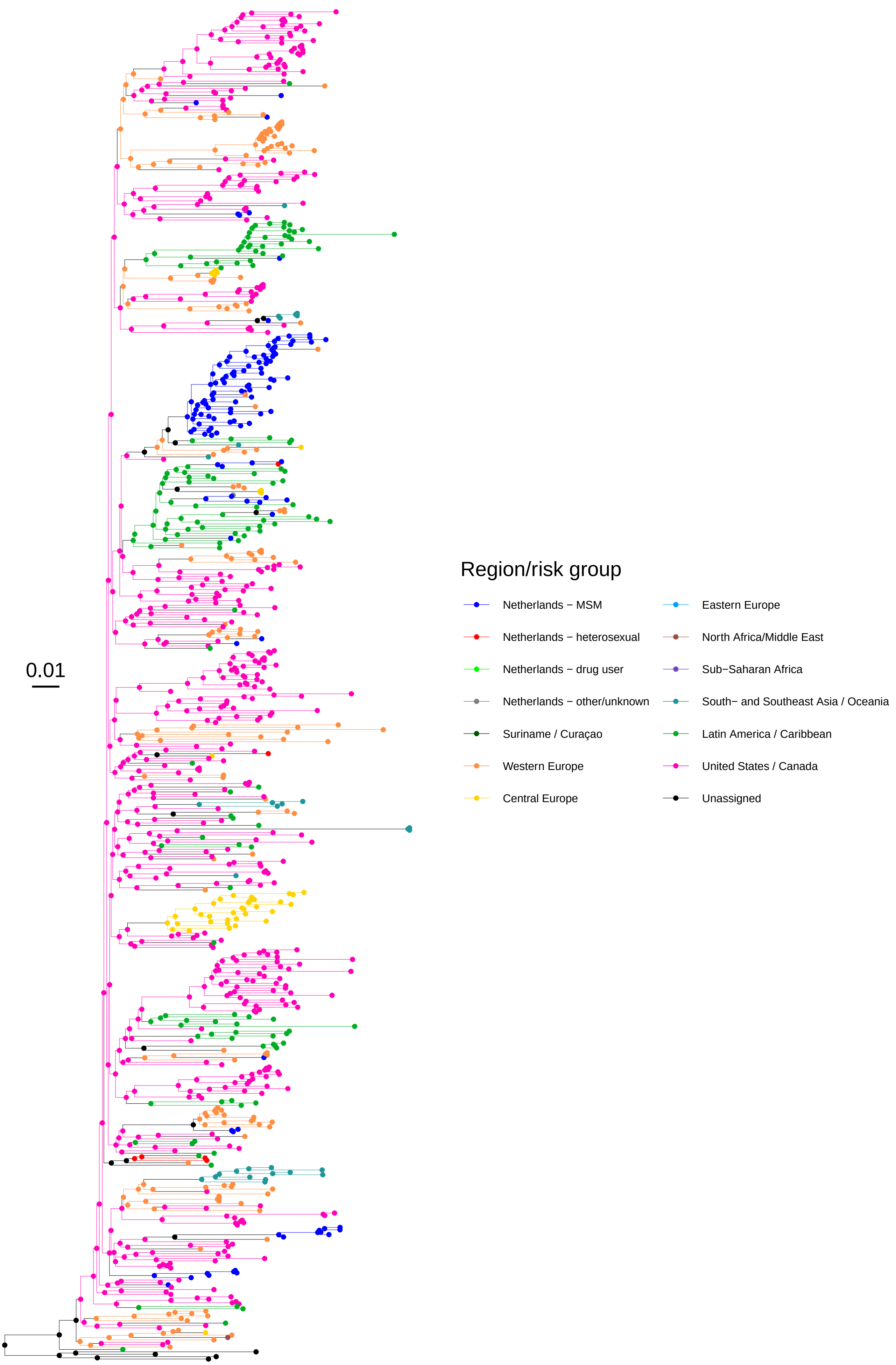
0.01
Region/risk group
Netherlands − MSM
Netherlands − heterosexual
Netherlands − drug user
Netherlands − other/unknown
Suriname / Curaçao
Western Europe
Central Europe
Eastern Europe
North Africa/Middle East
Sub−Saharan Africa
South− and Southeast Asia / Oceania
Latin America / Caribbean
United States / Canada
Unassigned

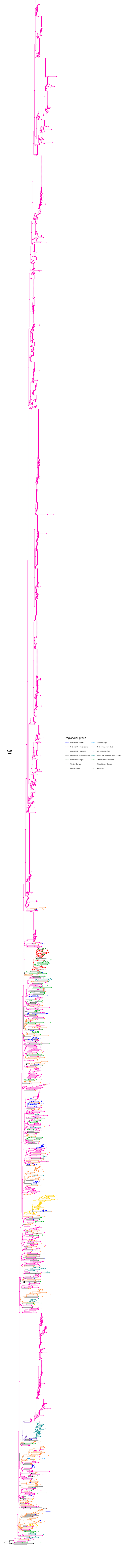

0.01
Region/risk group
Netherlands − MSM
Netherlands − heterosexual
Netherlands − drug user
Netherlands − other/unknown
Suriname / Curaçao
Western Europe
Central Europe
Eastern Europe
North Africa/Middle East
Sub−Saharan Africa
South− and Southeast Asia / Oceania
Latin America / Caribbean
United States / Canada
Unassigned

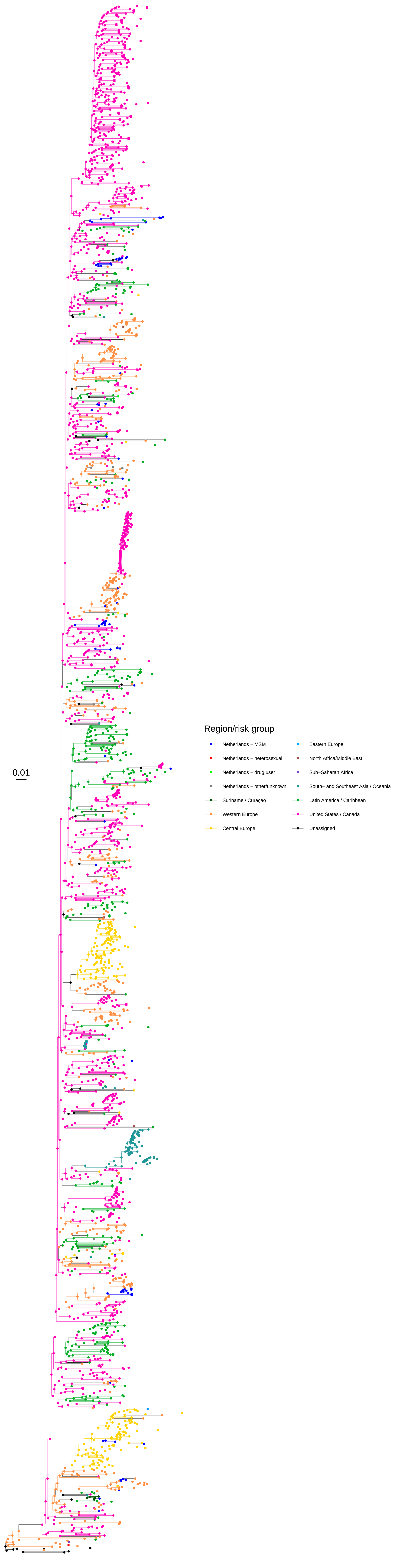
0.01
Region/risk group
Netherlands − MSM
Netherlands − heterosexual
Netherlands − drug user
Netherlands − other/unknown
Suriname / Curaçao
Western Europe
Central Europe
Eastern Europe
North Africa/Middle East
Sub−Saharan Africa
South− and Southeast Asia / Oceania
Latin America / Caribbean
United States / Canada
Unassigned
